# *In-silico* discovery of common molecular signatures for which SARS-CoV-2 infections and lung diseases stimulate each other, and drug repurposing

**DOI:** 10.1371/journal.pone.0304425

**Published:** 2024-07-18

**Authors:** Muhammad Habibulla Alamin, Md. Matiur Rahaman, Farzana Ferdousi, Arnob Sarker, Md. Ahad Ali, Md. Bayazid Hossen, Bandhan Sarker, Nishith Kumar, Md. Nurul Haque Mollah

**Affiliations:** 1 Faculty of Science, Department of Statistics, Bangabandhu Sheikh Mujibur Rahman Science and Technology University, Gopalganj, Bangladesh; 2 Zhejiang University-University of Edinburgh Institute, Zhejiang University School of Medicine, Zhejiang University, Haining, P. R. China; 3 Faculty of Science, Department of Biochemistry and Molecular Biology, University of Rajshahi, Rajshahi, Bangladesh; 4 Faculty of Science, Department of Statistics, Bioinformatics Laboratory (Dry), University of Rajshahi, Rajshahi, Bangladesh; 5 Faculty of Science, Department of Chemistry, University of Rajshahi, Rajshahi, Bangladesh; 6 Department of Agricultural and Applied Statistics, Bangladesh Agricultural University, Mymensingh, Bangladesh; Saveetha University - Poonamallee Campus: SIMATS Deemed University, INDIA

## Abstract

COVID-19 caused by SARS-CoV-2 is a global health issue. It is yet a severe risk factor to the patients, who are also suffering from one or more chronic diseases including different lung diseases. In this study, we explored common molecular signatures for which SARS-CoV-2 infections and different lung diseases stimulate each other, and associated candidate drug molecules. We identified both SARS-CoV-2 infections and different lung diseases (Asthma, Tuberculosis, Cystic Fibrosis, Pneumonia, Emphysema, Bronchitis, IPF, ILD, and COPD**)** causing top-ranked 11 shared genes (*STAT1*, *TLR4*, *CXCL10*, *CCL2*, *JUN*, *DDX58*, *IRF7*, *ICAM1*, *MX2*, *IRF9* and *ISG15*) as the hub of the shared differentially expressed genes (hub-sDEGs). The gene ontology (GO) and pathway enrichment analyses of hub-sDEGs revealed some crucial common pathogenetic processes of SARS-CoV-2 infections and different lung diseases. The regulatory network analysis of hub-sDEGs detected top-ranked 6 TFs proteins and 6 micro RNAs as the key transcriptional and post-transcriptional regulatory factors of hub-sDEGs, respectively. Then we proposed hub-sDEGs guided top-ranked three repurposable drug molecules (Entrectinib, Imatinib, and Nilotinib), for the treatment against COVID-19 with different lung diseases. This recommendation is based on the results obtained from molecular docking analysis using the AutoDock Vina and GLIDE module of Schrödinger. The selected drug molecules were optimized through density functional theory (DFT) and observing their good chemical stability. Finally, we explored the binding stability of the highest-ranked receptor protein RELA with top-ordered three drugs (Entrectinib, Imatinib, and Nilotinib) through 100 ns molecular dynamic (MD) simulations with YASARA and Desmond module of Schrödinger and observed their consistent performance. Therefore, the findings of this study might be useful resources for the diagnosis and therapies of COVID-19 patients who are also suffering from one or more lung diseases.

## 1. Introduction

The SARS-CoV-2 virus is responsible for COVID-19 which was the first outbreak in Wuhan city, Hubei province, China, in December 2019 [1]. Its outbreak became a terrible form rapidly whole over the world and the WHO officially announced it to be a global epidemic on March 11, 2020 [1]. Although COVID-19 has hurt almost all countries, notably the United States, India, France, Germany, Brazil, South Korea, and Japan are the top seven countries affected by SARS-CoV-2 (https://www.worldometers.info/coronavirus/). According to the WHO report, until December 31, 2023, over 6.9 million people out of 700 million SARS-CoV-2 infected people have died. Though infection rates are gradually decreasing worldwide due to the impact of vaccination, however, some people are yet infecting [2]. It may have happened due to the unstable RNA pattern of SARS-CoV-2 and the weak immunity of the patients. Not every patient with SARS-CoV-2 infection suffers in the same way. Some patients become more vulnerable, who are already suffering from one or more comorbidities like cardiovascular diseases [3], diabetes [4], hypertension [3], and different lung diseases including chronic obstructive pulmonary disease (COPD) [5], idiopathic pulmonary fibrosis (IPF) [6], interstitial lung disease (ILD) [7], asthma [8], tuberculosis [9], cystic fibrosis [4], pneumonia [1,10], emphysema [11], and bronchitis [12]. Chronic obstructive pulmonary disease (COPD) is a chronic inflammatory lung disorder that encompasses chronic bronchitis and emphysema, and it is characterized by restricted airflow with symptoms of breathing problems and cough with mucus due to abnormalities in the airways or air sacs of the lungs [13]. It is the third leading cause of death globally [14]. Idiopathic pulmonary fibrosis (IPF) is a long-term, progressive lung disorder characterized by lung scarring or fibrosis that leads to respiratory failure [15]. Patients with IPF survive only about 3–5 years after diagnosis with symptoms of dry cough and shortness of breath [16]. Respiratory failure is responsible for death related to IPF [16]. Interstitial lung disease (ILD) is a lung disorder that can stimulate both the vulnerability and severity of COVID-19 [7]. Tuberculosis (TB) constitutes the predominant cause of mortality associated with respiratory infections. Furthermore, TB significantly augments the susceptibility to COVID-19 while simultaneously exacerbating the severity of the disease [9]. People with cystic fibrosis (CF), a chronic lung disease involving mucus blockage and persistent airway inflammation, are highly vulnerable to COVID-19 due to the increased risk of severe viral respiratory infections [4]. Moreover, pneumonia is a type of lung disease characterized by inflammation in the tiny air sacs within the human lungs, leading to the accumulation of fluid and resulting in breathing difficulties [17].

**Fig 1:**
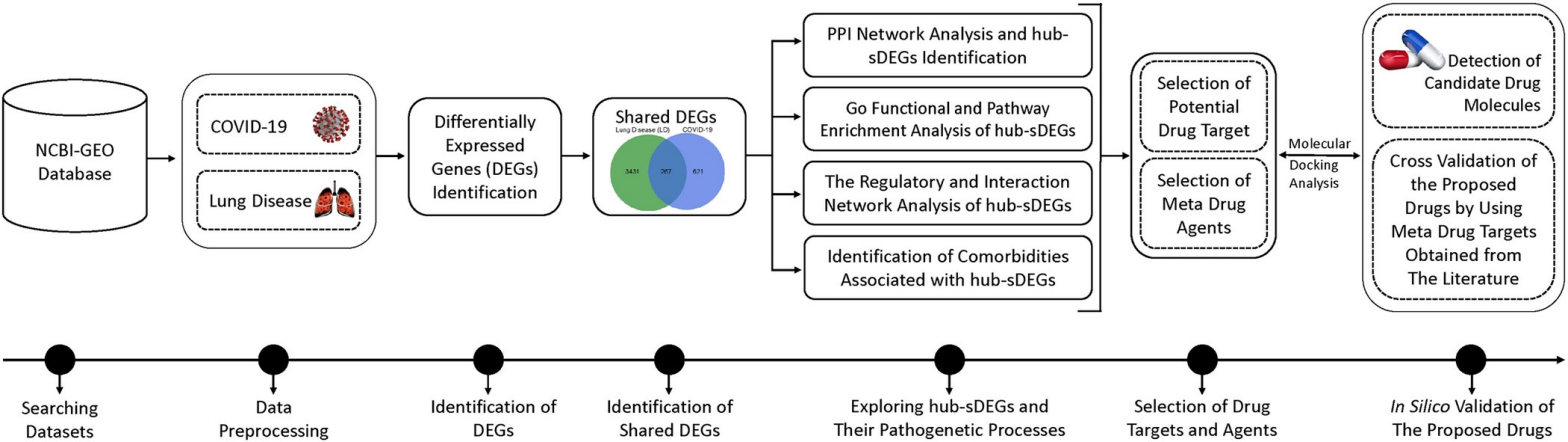
The complete workflow of this study.

The S-protein of SARS-CoV-2 has a higher interaction with ACE2 (angiotensin-converting enzyme 2); however, a significant amount of ACE2 is found in lung disease patients [13]. Therefore, in this study, we have considered chronic lung disease patients as a high-risk group for COVID-19 complications [7,13]. Several studies explored SARS-CoV-2 infections causing key genes (KGs). Some of these studies detected shared key-genes (sKGs) to disclose common pathogenetic processes of SARS-CoV-2 infections with one or two lung diseases including COPD [13], IPF [6], COPD and IPF [13], ILD [7], asthma [18], tuberculosis [19], cystic fibrosis [20], pneumonia [21], emphysema [13], and bronchitis [13]. Few of these studies recommended sKGs-guided common drug molecules in which molecules (curcumin, triclosan, tamoxifen, deguelin) were recommended for the treatment of SARS-CoV-2 infections with COPD [13], molecules (tegobuvir, nilotinib, digoxin, proscillaridin, simeprevir, sorafenib, torin 2, rapamycin, vancomycin and hesperidin) with IPF [6], molecules (suloctidil, estradiol, prenylamine, clioquinol) with asthma [18], molecules (rituximab, bevacizumab, bosentan, sitaxentan, and macitentana) with tuberculosis [19], molecules (imiquimod and raloxifene) with cystic fibrosis [20]. However, so far, there is no study that explored sKGs/sDEGs to disclose common pathogenetic mechanisms and associated drug molecules for SARS-CoV-2 infections and different lung diseases. Therefore, the main objective of this study is to explore potential sKGs/sDEGs to reveal the common pathogenetic mechanisms of SARS-CoV-2 infection and different lung diseases to adopt a common treatment plan. The workflow of this research is shown in **Fig 1**.

## 2. Materials and methods

### 2.1. Data sources and descriptions

In this study, we analyzed three RNA-Seq profile datasets for SARS-CoV-2 infections and nine microarray gene expression datasets for nine types of lung diseases (COPD, IPF, ILD, Asthma, Tuberculosis, Cystic Fibrosis, Pneumonia, Emphysema, and Bronchitis) that were collected from the Gene Expression Omnibus (GEO) platform of National Center for Biotechnology Information (NCBI) database [22]. The detail information of these datasets is given in **S1 Table**.

### 2.2.Identification of shared differentially expressed genes (sDEGs)

We considered three methods DESeq2, edgeR and LIMMA (voom) for the identification of DEGs between SARS-CoV-2 infections and control groups separately from RNA-Seq profile datasets [23,24]. To identify DEGs between lung disease and control samples based on microarray gene expression profile datasets, separately we utilized KW, SAM, and LIMMA approaches [25]. R-software (Version 4.0.5) has been used to implement these approaches for data analysis. Let $C_{i}^{DESeq2},C_{i}^{edgeR}$ and $C_{i}^{LIMMA(voom)}$ are three DEGs sets computed from *i*^th^ RNA-Seq count dataset by DESeq2, edgeR and LIMMA (voom), respectively, for COVID-19 (*i* = 1,2,3). Again, let $L_{j}^{KW},L_{j}^{SAM}$ and $L_{j}^{LIMMA}$ are three DEGs sets computed from microarray gene expression profiles by KW, SAM and LIMMA, respectively, for *j*^th^ lung disease (*j* = 1,2,…,9). Then we computed shared DEGs (sDEGs) between COVID-19 and different lung diseases as follows,

$$sDEGs=\left[({⋂}_{i=1}^{3}C_{i}^{DESeq2})\cup ({⋂}_{i=1}^{3}C_{i}^{edgeR})\cup ({⋂}_{i=1}^{3}C_{i}^{LIMMA(voom)})\right]\cap \left[{⋂}_{j=1}^{9}\left(L_{j}^{KW}\cup L_{j}^{SAM}\cup L_{j}^{LIMMA}\right)\right],$$ (1)

Since different methods (utilized in this study) identify DEGs based on different assumptions on expression data; however, a method fails to detect some potential DEGs when its assumption is not fully satisfied by the dataset [26].

### 2.3. Protein-protein interaction (PPI) network analysis

To construct protein-protein interaction (PPI) network, we inserted sDEGs in the ‘Search Tool for the Retrieval of Interacting Genes (STRING)’ database (version 11.5). Then, we used Cytoscape (version 3.10.0) software with a confidence score ≥ 0.90 to analyze and visualize the PPI network. We implemented a Cytoscape plugin Network Analyzer (version 4.4.8), to investigate the interactions among sDEGs. Then, we used the cytoHubba (version 0.1) plugin in Cytoscape to identify significant nodes or hub-sDEGs based on the degree scores [25,27].

### 2.4. Gene-disease interaction network analysis

To investigate the disease risk factors of SARS-CoV-2 infections through the hub-sDEGs, we performed ‘gene-disease’ interaction network analysis by using the web-tool ‘NetworkAnalyst (version 3.0)’ with the database ‘DisGeNET’ [28]. A disease has been considered as significantly associated with hub-sDEGs if adjusted *p*-value < 0.05.

### 2.5. GO functional and pathways enrichment analysis with hub-sDEGs

The GO functional and pathway enrichment analysis of hub-sDEGs were performed by using ‘Enrichr’ and ‘DAVID’ databases [6,24]. Then, we extracted common GO terms and pathways from both databases. The adjusted *p*-value < 0.05 was considered as the threshold value for this analysis.

### 2.6. The gene regulatory network (GRN) analysis of hub-sDEGs

To obtain key transcriptional and post-transcriptional regulators of hub-sDEGs, we performed network analysis of hub-sDEGs with TFs and miRNAs from TF2DNA and TarBase (v8.0) databases, respectively, and their interaction networks were constructed by using STRING (version 11.5) database and visualized in Cytoscape (version 3.10.0) software [24]. The significant key TFs and miRNAs were identified via the ‘CytoHubba’ plugin in Cytoscape based on the highest degree scores.

### 2.7. Hub-sDEGs guided drug repurposing by molecular docking studies

To explore hub-sDEGs mediated receptor-proteins guided few potential repurposable drug molecules for the treatment against SARS-CoV-2 infections with one or more lung diseases by molecular docking analysis, a total number of ***n*** = 184 candidate drug molecules (**S2 Table**) were accumulated from different published articles associated with SARS-CoV-2 infections and/or different lung diseases. Before going to the molecular docking analysis, both the receptor-proteins and drug/ligand structures were prepared for molecular docking. The crystallographic structure of the target protein was obtained from the Protein Data Bank (PDB) and AlphaFold databases [6]. The ‘PubChem’ database was used to retrieve the 3D structures of those meta-drug agents. To identify potential binding sites within the target protein’s catalytic site (target pocket), we employed the ‘PrankWeb’ tools which employs a template-free machine learning method called ‘P2Rank’ for predicting ligand binding sites on solvent-accessible protein surfaces and identifying receptor protein target pockets [29]. Then, ‘PyMOL’ was used to visualize and select amino acid residues for the active sites of target proteins [24].

#### 2.7.1. Molecular docking using autodock vina

AutoDock tools 1.5.7 were utilized to process the receptor-proteins by removing water molecules, adding charges, and centering the grid box on the active site with specific coordinates [24]. The drug agents/ligands were pre-processed by minimized energy through the ‘Avogadro’ software and setting torsion tree using ‘AutoDock tools 1.5.7’. Both prepared receptor and ligands were converted into the PDBQT format. Subsequently, using the ‘AutoDock Vina’ [24], the binding affinities between the drug agents and target proteins were determined. Let $A_{ij}$ be the binding affinity score (BAS) between *i*^*th*^ target protein (*i* = 1,2,…,*m*) and *j*^*th*^ drug agent (*j* = 1,2,…,*n*). The target proteins and drug agents were arranged according to the descending order of row sums $\sum_{j=1}^{n}A_{ij};\;i=1,\ldots ,m$, and column sums $\sum_{i=1}^{m}A_{ij};\;j=1,\ldots ,n,$ respectively, to pick the best-performing drugs as the candidate drugs. Then, we constructed the image of BAS corresponding to the ordered drugs and targets using TBtools [30]. Finally, the discovery studio visualizer (v21.1.0.0) and ‘PyMOL’ were used to display receptor-ligand interactions involving amino acids and their interactive position in the docked complex molecule.

#### 2.7.2. Molecular docking through Schrödinger software

At first, the ligands were prepared by utilizing LigPrep module of Schrödinger [31] which involves the minimization of the appropriate bond angles and using force fields OPLS3 to minimize the structure’s energy. Next, several steps were performed to prepare proteins using protein preparation wizard tools. These steps involve adding hydrogen, utilizing prime to complete side chain gaps, replacing any absent loops with prime, removing water molecules located farther than 5.00 angstroms from the specified HET group, and producing the protonation state of heteroatoms using Epik, with a pH range of 7.0 ± 2.0. Finally, docking analysis was performed between the active site of the proteins and the drugs.

### 2.8. Optimization of drug compounds and their chemical reactivity calculation

Drug compounds were optimized using Gaussian 09 [32], a powerful computational chemistry software suite renowned for performing geometry optimizations and electronic structure calculations. The resulting data were then visualized and analyzed using GaussView 05 [33], a user-friendly interface that seamlessly integrates with ‘Gaussian 09’. Key parameters such as the highest occupied molecular orbital (HOMO) energies, lowest unoccupied molecular orbital (LUMO) energies, electron affinity, and electrophilicity index were calculated. These parameters play a crucial role in explaining the magnitude of ligand interaction in the binding pocket of the receptor protein.

### 2.9. Molecular dynamic (MD) simulations

To evaluate the stability and flexibility of the top-ranked protein-ligand complexes, we considered two software’s (YASARA and Desmond) for molecular dynamics (MD) simulation for a duration of 100 nanoseconds.

#### 2.9.1. Molecular dynamic simulations using YASARA

To investigate the dynamic behavior of the top-ranked protein-ligand complexes, MD simulations were performed using the AMBER14 force field by the YASARA Dynamics software [34,35]. Before starting the simulation, the hydrogen bonding network of the target-drug combination was solvated and tuned by a TIP3P water model [36]. Using the steepest gradient approach (5000 cycles), a simulated annealing method was used for the initial energy minimization of each simulation system. A 100 ns MD simulation was carried out under a Berendsen thermostat and constant pressure [37]. The YASARA macro’s default script and SciDAVis (http://scidavis.sourceforge.net/) were used for this analysis. Then, using the following formula.

BindingfreeEnergy=EpotReceptor+EsolvReceptor+EpotLigand+EsolvLigand−EpotComplex−EsolvComplex

the binding free energy of each snapshot was calculated by the molecular mechanics Poisson–Boltzmann surface area (MM-PBSA) function of the YASARA software.

#### 2.9.2. Molecular dynamic simulations using Desmond module of Schrödinger

Initially, the Schrödinger software’s system constructor wizard was used to solvate the protein-ligand complex (PLC) with water molecules [38]. The study employed a transferable intermolecular potential 3P (TIP3P) solvent system [39]. The computational analyses were conducted within an orthorhombic box of 10 × 10 × 10 Å, utilizing periodic boundary conditions. A constant salt concentration of 0.15 M was maintained. The entire model system was subjected to a 100 ps minimization process, and the resulting trajectory was utilized for conducting a molecular dynamics investigation. The dynamics investigations were conducted for a duration of 100 nanoseconds, with a recording interval of 50 picoseconds. This resulted in a total of 5000 frames for the whole study. The simulations utilized a time step of 2 femtoseconds. The binding free energy of each snapshot in the protein-ligand interactions during high-throughput molecular dynamics (MD) simulations was calculated with MM-GBSA using gmx_MM-PBSA tools. The binding free energy (Δ*G*_*bind*_) was calculated by using the following equation:

$${\Delta G}_{bind}=E_{complex}-(E_{protein}+E_{ligand})$$

This comprehensive approach encompasses multiple interaction-free energies, including van der Waals forces, electrostatic interactions, polar solvation effects, solvent-accessible surface area (SASA) contributions, and binding energies. In this study, we have employed g_mmpbsa package [40,41]. The trajectories from the protein-ligand MD simulation in explicit water from the Desmond module were used to generate the GROMACS trajectory file required for calculations using Schrödinger scripts. Additionally, topology files for protein and ligand were obtained separately by converting the *.cms files to *.gro and *.top files using the InterMol software [42].

## 3. Results

### 3.1. Identification of shared differentially expressed genes (sDEGs)

Three RNA-Seq count datasets (GSE147507, GSE150392, and GSE152075) were analyzed by using three methods DESeq2, edgeR, and LIMMA (voom), and detected a total of 888 shared DEGs between COVID-19 and control samples as

$$\left[({⋂}_{i=1}^{3}C_{i}^{DESeq2})\cup ({⋂}_{i=1}^{3}C_{i}^{edgeR})\cup ({⋂}_{i=1}^{3}C_{i}^{LIMMA(voom)})\right]=888$$

Then, we analyzed GSE64913, GSE34608, GSE107846, GSE42830, GSE1122, GSE22148, GSE53845, GSE40839, and GSE100281 datasets for Asthma, Tuberculosis, Cystic Fibrosis, Pneumonia, Emphysema, Bronchitis, IPF, ILD, and COPD, respectively using three methods KW, SAM and LIMMA and combinedly identified total 3698 DEGs for these lung diseases as

$$\left[{⋂}_{j=1}^{9}\left(L_{j}^{KW}\cup L_{j}^{SAM}\cup L_{j}^{LIMMA}\right)\right]=3698$$

**Fig 2:**
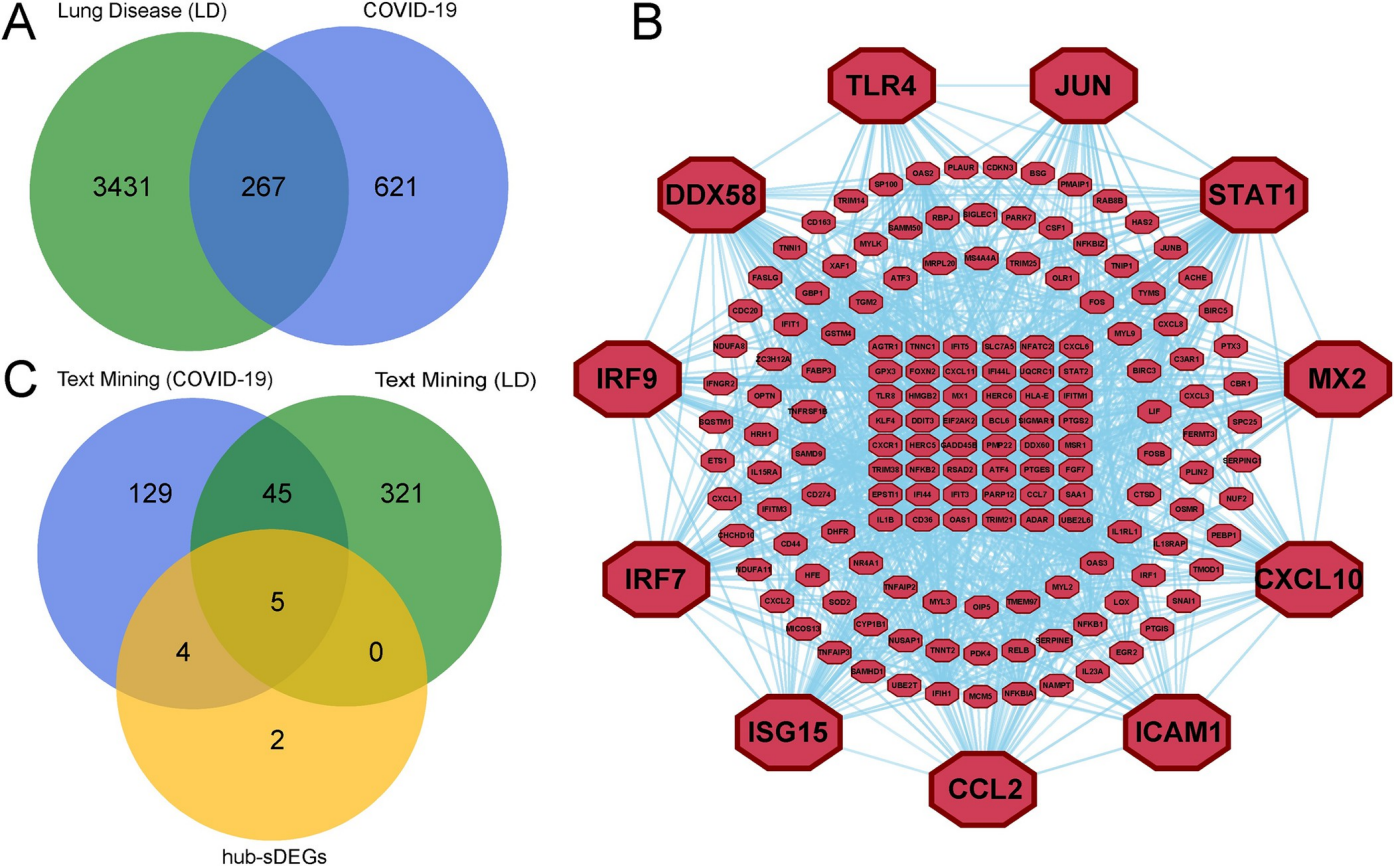
(A) Venn diagram of DEGs for COVID-19 and lung diseases to display shared DEGs (sDEGs). The blue and green circles represent DEGs from three datasets of COVID-19 and nine datasets of lung diseases, respectively. (B) Protein-protein interaction network of 267 sDEGs, where the large size maroon color octagonal node indicates hub-sDEGs. (C) Venn diagram among hub-sDEGs, reviewed DEGs of COVID-19, and reviewed DEGs of lung diseases.

Then we found 267 shared DEGs (sDEGs) between COVID-19 and different lung diseases using Eq 1 (**Fig 2A)**.

### 3.2. Identification of hub-sDEGs through PPI network analysis

The PPI network of 267 sDEGs was constructed which contained 267 nodes and 291 edges displayed in **Fig 2B** to detect the biomarker genes named hub-sDEGs. Based on the degree of importance, we selected the top-ranked 11 genes: *STAT1*, *TLR4*, *CXCL10*, *CCL2*, *JUN*, *DDX58*, *IRF7*, *ICAM1*, *MX2*, *IRF9* and *ISG15* as the hub-sDEGs and used for further analysis. Among them, 5 genes (*CCL2*, *CXCL10*, *ICAM1*, *JUN*, *TLR4*) are common with text mining of COVID-19 and lung disease presented through the Venn diagram in **Fig 2C**.

### 3.3. Association of hub-sDEGs with different diseases

**Fig 3:**
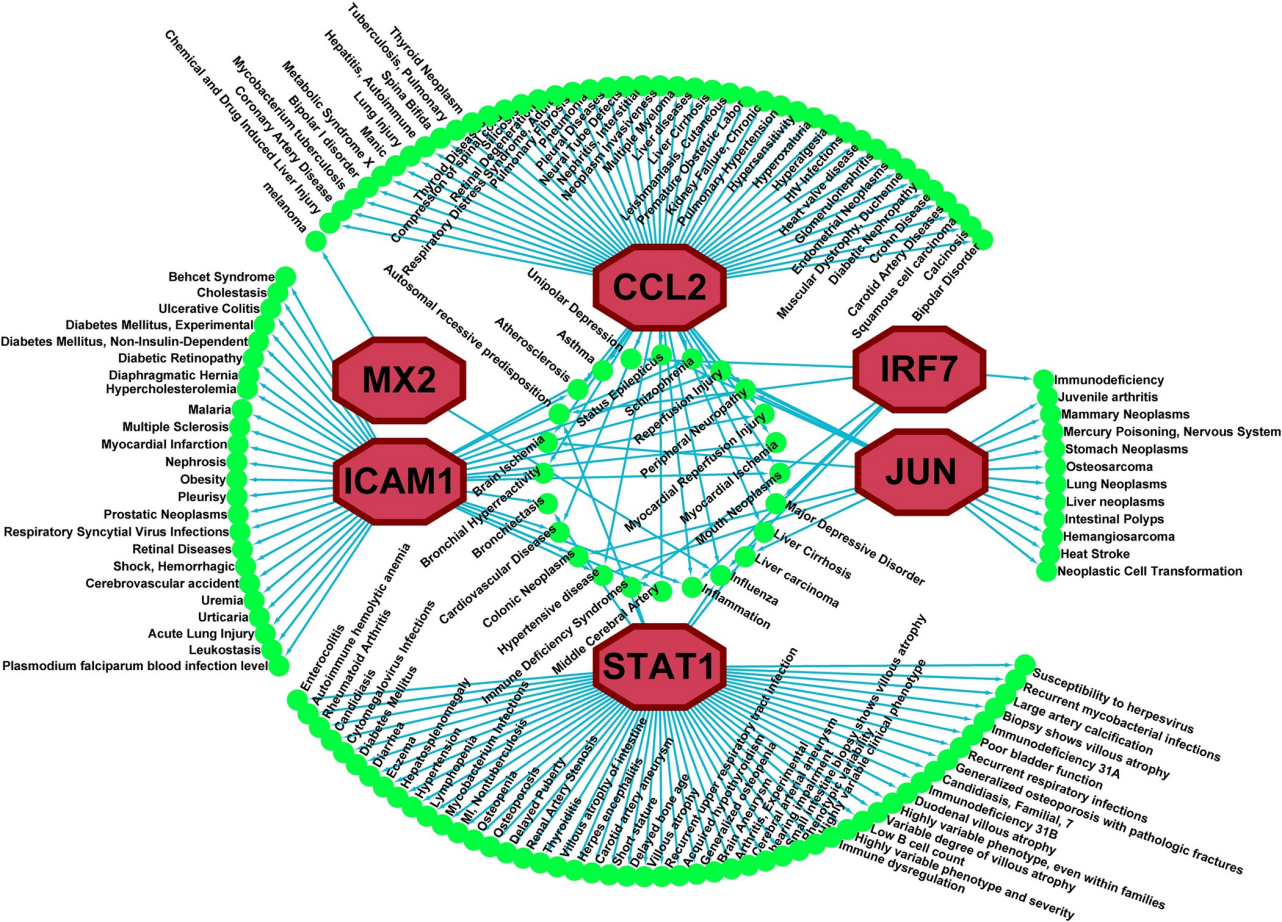
The disease *vs*. hub-sDEGs interaction network represents the disease risk factors of SARS-CoV-2 infections. Here the green circle node indicates significant disease risk and the maroon-colored octagonal node indicates hub-sDEGs.

To investigate the different disease risk factors of SARS-CoV-2 infections from the genetic viewpoint, the interaction network analyses of hub-sDEGs with different diseases were performed which revealed that 6 hub-sDEGs (*ICAM1*, *MX2*, *CCL2*, *IRF7*, *JUN*, *STAT1*) out of 11 are significantly associated with different diseases including asthma, atherosclerosis, bronchiectasis, cardiovascular diseases, brain ischemia, diabetes mellitus, obesity, schizophrenia, urticaria, melanoma, pneumonia, pulmonary fibrosis, tuberculosis, lung injury, lung neoplasms, rheumatoid arthritis, liver cirrhosis, hypertension, etc. (**Fig 3** and **S3 Table)**.

### 3.4. GO functions and pathways enrichment analysis of hub-sDEGs

GO functions are classified into three subsections: biological processes (BPs), molecular functions (MFs), and cellular components (CCs). The few top common enriched GO functions for each of the three subsections (BPs, MFs, and CCs) based on two online databases (DAVID and Enrichr) are presented in **Table 1**. According to the BP-terms, almost all the hub-sDEGs were mainly enriched with the defense response to virus, positive regulation of interferon-alpha/beta production, positive regulation of transcription by RNA polymerase II, positive regulation of DNA-templated transcription and cellular response to type II interferon, etc. Among the enriched CC-terms, endosome membrane, cytoplasmic vesicle membrane, nucleus, euchromatin, and intracellular membrane-bounded organelle are the top enriched GO functions. In the case of MFs, hub-sDEGs are significantly enriched in chemokine activity, transcription regulatory region sequence-specific DNA binding, RNA polymerase II core promoter proximal region sequence-specific DNA binding, double-stranded DNA binding, etc. Pathway enrichment analysis of the hub-sDEGs based on four databases (KEGG, WikiPathways, Reactome, and BioCarta) identified some crucial pathways (**S4 Table)**. Top-ranked four pathways (Coronavirus disease, Interferon signaling pathway, Immune responses, and Measles virus infection) supported by at least two databases are shown in **Table 2.**

**Table 1:** Significantly enriched common GO-terms (BPs, MFs, and CCs) that might be associated with SARS-CoV-2 infections and some lung diseases identified from two online web-tools DAVID and Enrichr (adjusted *p*-value < 0.05).

GO Category	GO ID	GO-terms	Associated hub-sDEGs
**Biological Process**	GO:0051607	defense response to virus	CXCL10; STAT1; MX2; IRF7; ISG15
	GO:0032727	positive regulation of interferon-alpha production	STAT1; IRF7; TLR4
	GO:0032728	positive regulation of interferon-beta production	IRF7; ISG15; TLR4
	GO:0045944	positive regulation of transcription by RNA polymerase II	CXCL10; JUN; STAT1; IRF7; TLR4; IRF9
	GO:0071346	cellular response to interferon gamma	STAT1; CCL2; TLR4
	GO:0045893	positive regulation of DNA-templated transcription	CXCL10; JUN; STAT1; IRF7; TLR4; IRF9
**Cellular Component**	GO:0010008	Endosome Membrane	IRF7; TLR4
	GO:0030659	Cytoplasmic Vesicle Membrane	IRF7; TLR4
	GO:0005634	Nucleus	JUN; STAT1; MX2; IRF7; ISG15; IRF9
	GO:0000791	Euchromatin	JUN
	GO:0043231	Intracellular Membrane-Bounded Organelle	JUN; STAT1; MX2; IRF7; ISG15; IRF9
**Molecular Function**	GO:0003690	double-stranded DNA binding	JUN; STAT1; IRF7; IRF9
	GO:0008009	chemokine activity	CXCL10; CCL2
	GO:0044389	ubiquitin-like protein ligase binding	JUN; STAT1; ISG15
	GO:0000978	RNA polymerase II core promoter proximal region sequence-specific DNA binding	JUN; STAT1; IRF7; IRF9
	GO:1990837	sequence-specific double-stranded DNA binding	JUN; IRF7; IRF9
	GO:0000976	transcription regulatory region sequence-specific DNA binding	JUN; STAT1

**Table 2:** Summary table of the significantly enriched common pathways with hub-sDEGs that might be associated with SARS-CoV-2 infections and some lung diseases identified from two online web-tools DAVID and Enrichr (adjusted *p*-value < 0.05).

Common pathways	Associated hub-sDEGs in different databases			
Pathways	KEGG	BioCarta	Reactome	WikiPathways
Coronavirus disease	✓	-	-	✓
Interferon signaling pathway	-	✓	✓	✓
Immune responses	-	-	✓	✓
Measles virus infection	✓	-	-	✓

### 3.5. The gene regulatory network (GRN) analysis of hub-sDEGs

**Fig 4:**
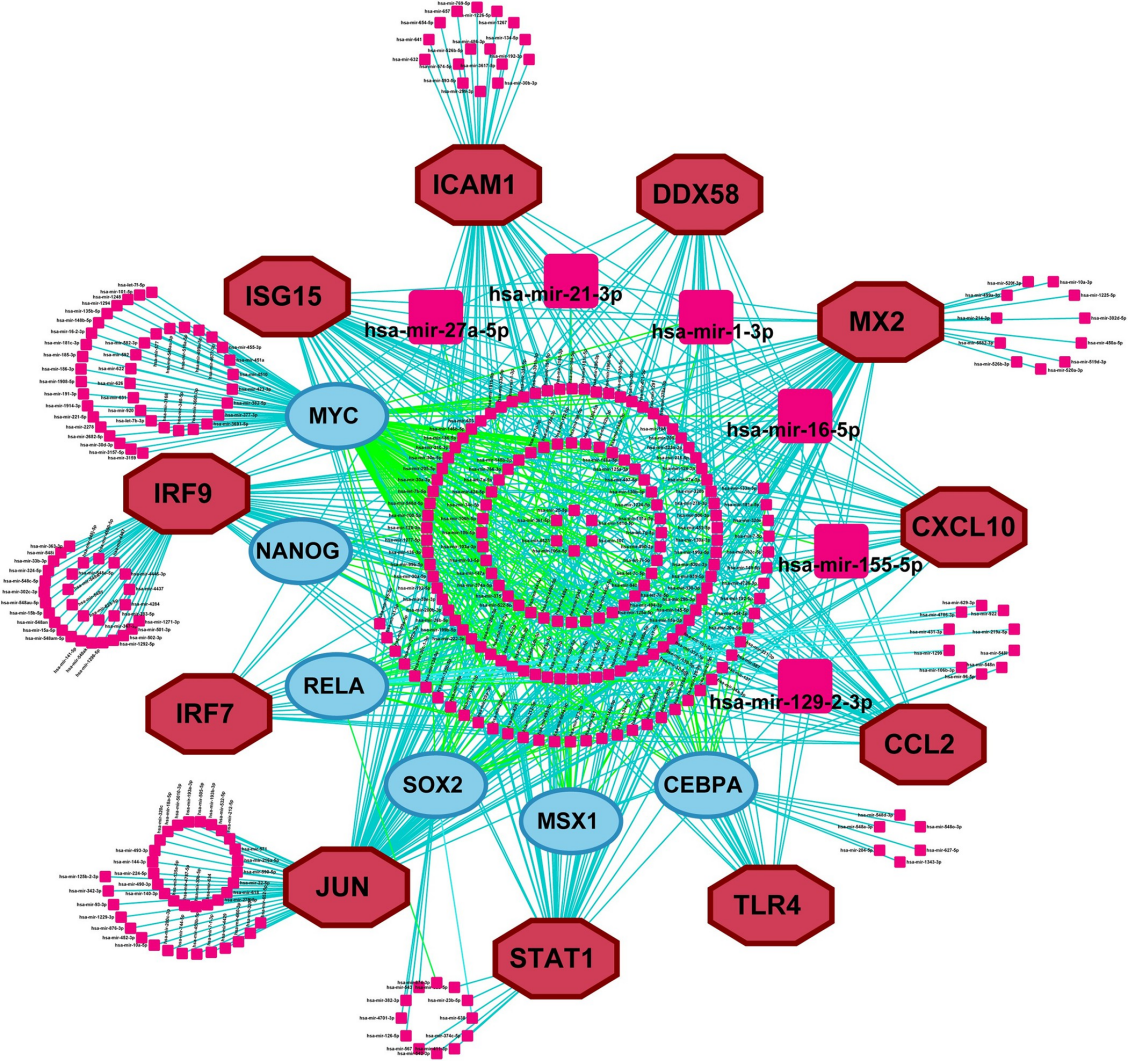
The miRNAs, TFs, and hub-sDEGs interaction network where the pink color round square node indicates miRNAs, the sky-blue color ellipse node indicates TFs and the maroon color octagonal node indicates hub-sDEGs. The key miRNAs are indicated by larger round square nodes.

To identify key transcriptional and post-transcriptional regulatory factors of hub-sDEGs, we constructed the interaction network among miRNAs, TFs, and hub-sDEGs as depicted in **Fig 4**. From this network, we selected top-ranked six significant TFs proteins (MYC, SOX2, CEBPA, NANOG, RELA, and MSX1) and six significant miRNAs (hsa-miR-16-5p, hsa-miR-129-2-3p, hsa-miR-21-3p, hsa-miR-27a-5p, hsa-miR-1-3p, hsa-miR-155-5p) as the key transcriptional and post-transcriptional regulatory factors of hub-sDEGs, based on the degree score.

### 3.6. Drug repurposing by molecular docking studies

**Fig 5:**
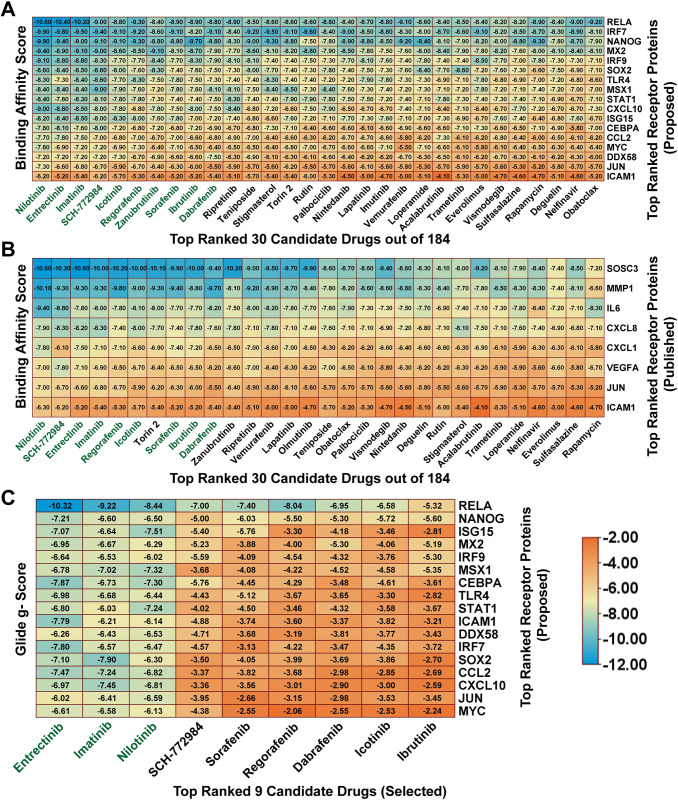
Matrix plot of the binding score obtained through the molecular docking analysis. (A) Image of BAS of the top-ranked 30 candidate drugs out of 184 meta-drug agents in the X-axis with the proposed top-ranked 17 drug target proteins in the Y-axis. (B) Image of BAS of the top-ranked 30 candidates published drugs in the X-axis against the published top-ranked 8 independent meta receptors in the Y-axis. (C) Image of Glide g-score of the top-ranked selected nine candidate drugs in the X-axis with the proposed top-ranked 17 drug target proteins in the Y-axis.

To explore candidate repurposable drugs as a therapy for SARS-CoV-2 infections with one or more lung diseases, we choose our suggested 11 hub-sDEGs based proteins (STAT1, TLR4, CXCL10, CCL2, JUN, DDX58, IRF7, ICAM1, MX2, IRF9, and ISG15) and its regulatory 6 key TFs proteins (MYC, SOX2, CEBPA, NANOG, RELA, and MSX1) as the *m* = 17 target proteins and 184 meta-drug agents (ligands). The 3D structures of 11 receptor proteins (CCL2, DDX58, ICAM1, ISG15, JUN, MYC, NANOG, RELA, SOX2, STAT1, TLR4) were downloaded from the PDB with source code (1dok, 2lwd, 2oz4, 3phx, 1jun, 1nkp, 2kt0, 4kv1, 2le4, 3wwt, 3fxi) and remaining 6 receptor proteins (CEBPA, CXCL10, IRF7, IRF9, MSX1, MX2) were obtained from AlphaFold source using Uniprot IDs P49715, P02778, Q92985, Q00978, P28360, P20592. Then molecular docking analyses were performed between *m* = 17 drug target proteins and *n* = 184 drug agents to obtain the binding affinity score (kcal/mol) for each target protein with each drug agent (**S5 Table)**. **Fig 5A** represents the ordered top-ranked 30 drugs binding affinity score matrix out of 184 drugs. We observed that the first four top-ordered compounds (Nilotinib, Entrectinib, Imatinib, and SCH-772984) produce highly significant binding affinity scores (≤ -7) kcal/mol with at least 14 receptor proteins out of 17 and their average binding affinity score is less than or equal to −8.0 kcal/mol. The remaining six top-ordered compounds (Icotinib, Regorafenib, Zanubrutinib, Sorafenib, Ibrutinib, and Dabrafenib) also produced significant binding with at least 12 receptors out of 17, and their average binding affinity score (≤ −7.4) kcal/mol. Hence, we considered these top-ranked 10 compounds (Nilotinib, Entrectinib, Imatinib, SCH-772984, Icotinib, Regorafenib, Zanubrutinib, Sorafenib, Ibrutinib, and Dabrafenib) as the most viable candidate drugs to treat COVID-19 as well as patients with lung diseases and highlighted them in **Fig 5A.** To investigate the resistance performance of the considered drug molecules, and compare to the already published molecules against the state-of-the-arts alternatives top-ranked independent receptors published by other studies, we reviewed 27 published articles related to COVID-19 (**S6 Table**) and 27 published articles associated with lung diseases (**S7 Table**). Then, we selected eight top-ranked target proteins that are commonly reported in at least two articles in both SARS-CoV-2 and lung disease-related literature and highlighted them in the 5th column in **S6** and **S7 Tables**. These 8 target proteins were considered as the top-ranked independent meta-receptors to examine the resistivity of the considered drugs compared to the selected top-ranked published drugs by molecular docking analysis. To examine the resistivity of the considered 10 candidate drugs against the top-ranked eight independent meta receptors, we downloaded the 3D structures of the seven (ICAM1, JUN, MMP1, CXCL8, CXCL1, VEGFA, and IL6) independent meta receptor proteins from the PDB with source codes 2oz4, 1jun, 1ayk, 1o7b, 1msh, 1kat, and 1il6, respectively and the protein SOSC3 downloaded from the AlphaFold database using Uniprot ID O14543. Then, we performed molecular docking analysis of published drugs with the top-ranked 8 independent meta-receptor proteins, and their docking results are given in **Fig 5B and S8 Table**. We observed that among the considered drugs, 9 drugs (Nilotinib, Entrectinib, Imatinib, SCH-772984, Icotinib, Regorafenib, Sorafenib, Ibrutinib, and Dabrafenib) belong to the top-ranked ten drugs against the independent receptors also. Hence, we selected these nine drugs as the potential drugs for the therapy of SARS-CoV-2 infections and lung diseases. To verify the significance of the protein-ligand interaction for our proposed target proteins and the selected ligands (drugs), we also took into account an alternative docking tool known as the GLIDE module of Schrödinger and calculated binding scores (Glide g-score, Docking score, and Glide e-model) (**Fig 5C** and **S9–S11 Tables**).

### 3.7. Optimization of drug compounds and their chemical reactivity calculation

Selected nine compounds from molecular docking analysis were further optimized through density functional theory (DFT). For that, Frontier molecular orbitals (FMO) properties (such as HOMO and HUMO) of nine compounds were computed to determine the significance of charge-transfer interactions at the protein binding site (**Table 3**).

**Table 3:** Physio-chemical descriptors and their reactivity descriptor analysis of the top-ranked nine compounds.

	Nilotinib	Entrectinib	Imatinib	SCH- 772984	Icotinib	Regorafenib	Sorafenib	Ibrutinib	Dabrafenib
**ԑHOMO**	-0.219	-0.192	-0.198	-0.190	-0.203	-0.226	-0.224	-0.212	-0.224
**ԑLUMO**	-0.078	-0.033	-0.053	-0.050	-0.047	-0.047	-0.043	-0.042	-0.069
**Energy gap** Δԑ = ԑLUMO–ԑHOMO)	0.141	0.159	0.145	0.140	0.156	0.179	0.181	0.170	0.155
**Ionization potential** (I = ˗ԑHOMO)	0.219	0.192	0.198	0.190	0.203	0.226	0.224	0.212	0.224
**Electron affinity** (A = ˗ԑLUMO)	0.078	0.033	0.053	0.050	0.047	0.047	0.043	0.042	0.069
**Electro-negativity** (χ = (I+A)/2)	0.149	0.113	0.125	0.120	0.125	0.137	0.134	0.127	0.146
**Chemical potential** (μ = ˗(I+A)/2)	-0.149	-0.113	-0.125	-0.120	-0.125	-0.137	-0.134	-0.127	-0.146
**Chemical hardness** (ղ = (I-A)/2)	0.070	0.079	0.072	0.070	0.078	0.090	0.090	0.085	0.077
**Electrophilicity index** (ω = μ^2^/2η)	0.157	0.080	0.108	0.104	0.100	0.104	0.099	0.095	0.138
**Softness** (S = 1/ղ)	6.358	12.520	9.233	9.658	9.965	9.611	10.093	10.567	7.230

### 3.8. Molecular dynamic (MD) simulations

**Fig 6:**
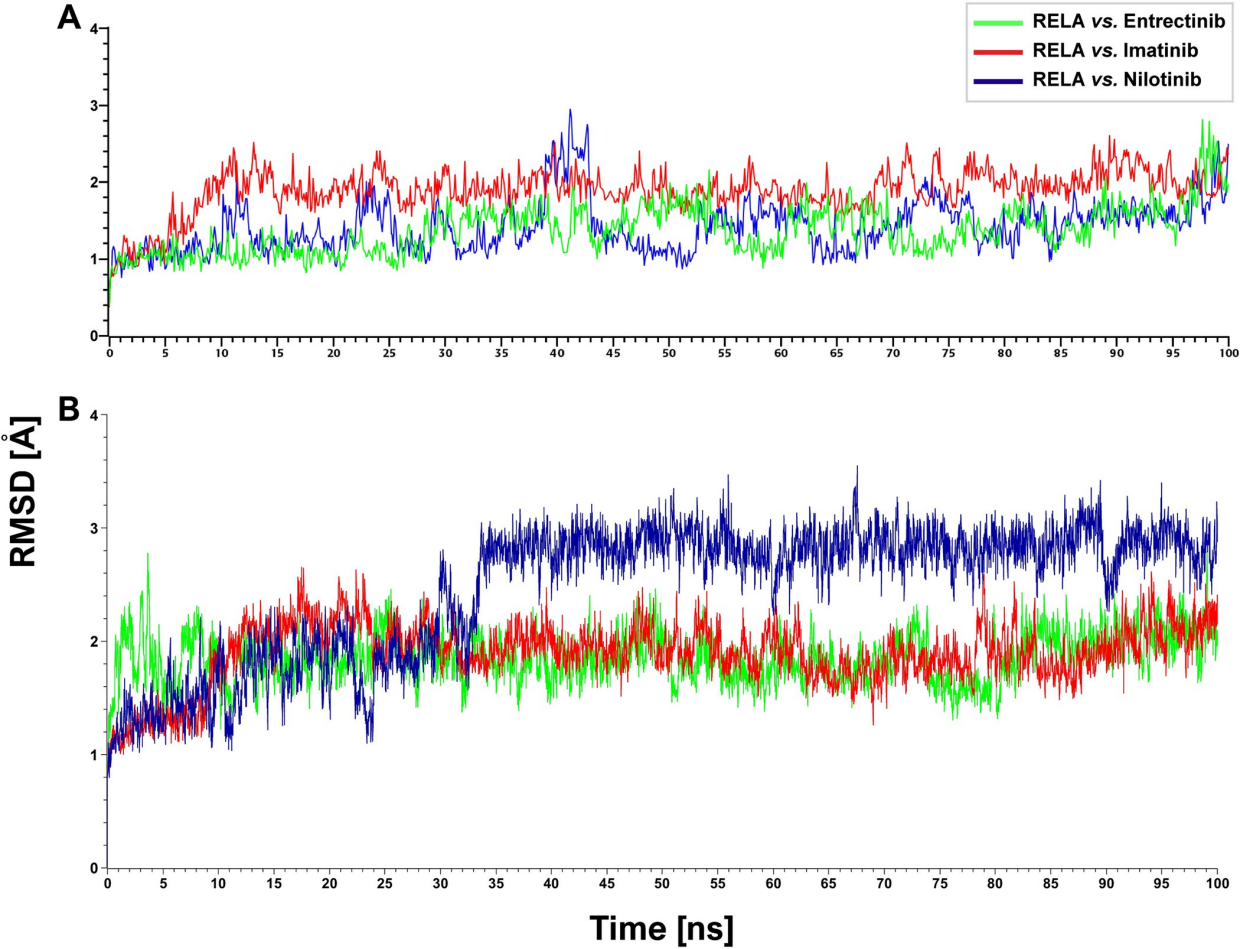
The root mean square deviation (RMSD) analysis results for a duration of 100 ns simulation with each of the top-ranked three drug-target complexes. (A) The RMSD analysis results with YASARA. (B) The RMSD analysis results with the Desmond module of Schrödinger.

Among the selected candidate drugs—Nilotinib, Entrectinib, and Imatinib were the top-ranked three candidate drugs, and we choose RELA for MDS, since RELA *vs*. Nilotinib, RELA *vs*. Entrectinib, and RELA *vs*. Imatinib complexes produce highest binding affinity scores compare to the other complexes (**Fig 5**). Therefore, these top three drug-target complexes were selected for their stability analysis with the molecular dynamics (MD) simulation for a duration of 100 nanoseconds. To perform MD simulation using YASARA, the initial energy minimization of each simulation system included 33769, 33564, and 44604 atoms for the RELA *vs*. Nilotinib, RELA *vs*. Entrectinib, and RELA *vs*. Imatinib complexes, and the average RMSDs for these systems were 1.42 Å, 1.37 Å, and 1.89 Å, respectively. These three complexes were almost stable throughout the whole simulation and all of the systems projected RMSD between 0.36 Å and 2.95 Å, though in the case of RELA *vs*. Nilotinib complex, a steady movement was found from 38 ns to 43 ns (**Fig 6A**). To examine the stability of these three complexes by another software, we considered the Desmond module of Schrödinger. The conformational stability of biological molecules were investigated using the Root Mean Square Deviation (RMSD) [43]. The protein backbone RMSDs were used to assess the deviation of the RELA protein bound with proposed inhibitors. The RMSD values of RELA for each frame computed through MD simulation production bound with all three drug molecules were plotted against the time scale, as shown in **Fig 6B**, which shows that the three complexes RELA *vs*. Entrectinib, RELA *vs*. Imatinib, and RELA *vs*. Nilotinib average RMSDs were 1.85 Å, 1.89 Å, and 2.47 Å, respectively. These results indicated that the complexes of Entrectinib and Imatinib with RELA are almost stable throughout the whole simulation and all of the systems projected an RMSD between 1.0 Å and 2.75 Å. However, the RELA *vs*. Nilotinib complex shows a slight fluctuation than the other two complexes between 0 to 35 ns, after reaching at 40ns, it seems quite stable and no complex has the RMSD greater than 3.0 Å. Thus, in the case of RMSD, it is clearly observed from both software that none of those three complexes has more fluctuation and they formed stable interactions.

**Fig 7:**
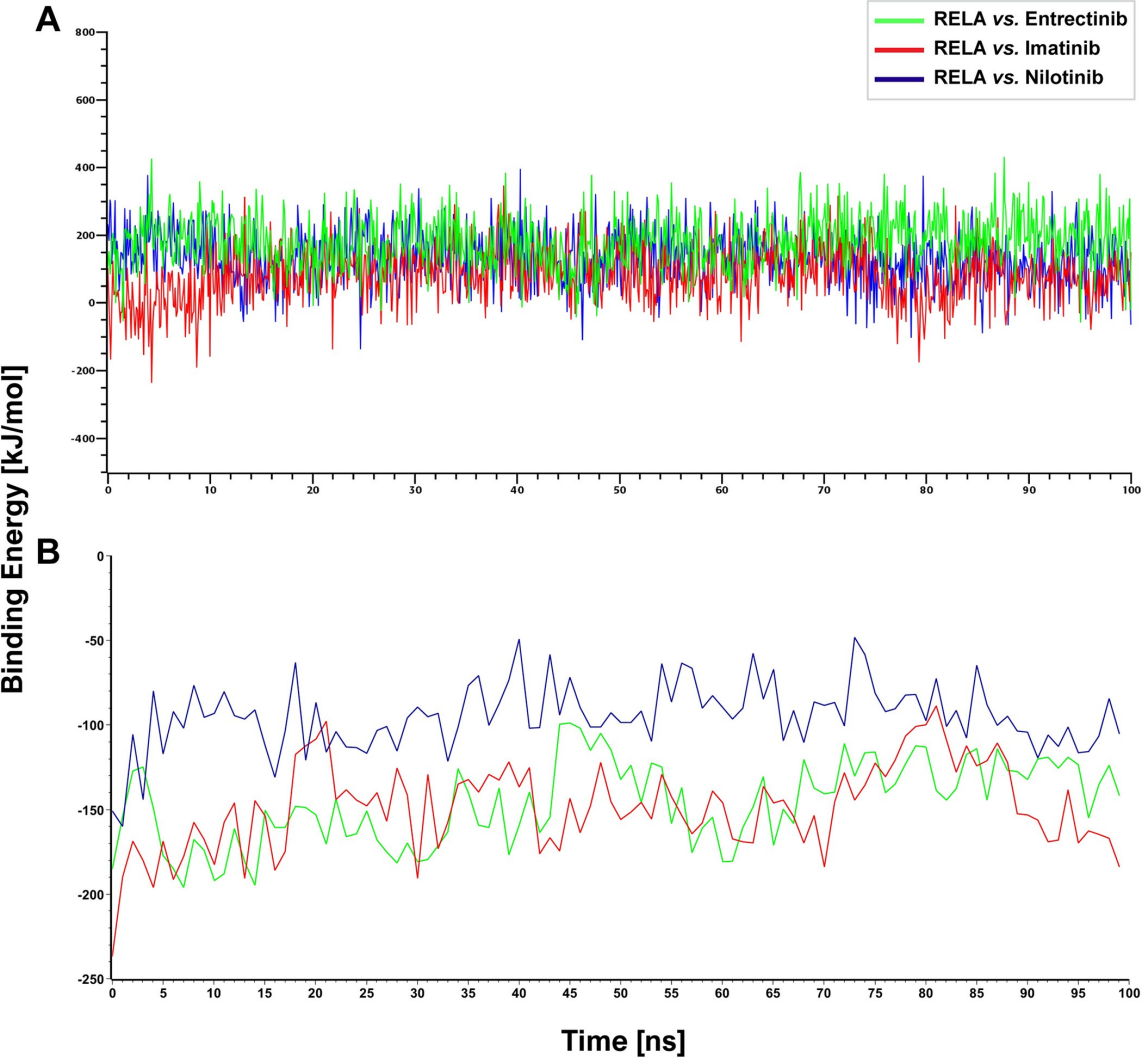
Binding energies (kJ/mol) for a duration of 100 ns simulation with each of the top-ranked three drug-target complexes. (A) The MM-PBSA binding energies for the MD simulation with YASARA (B) The MM-GBSA binding energies for the MD simulation with the Desmond module of Schrödinger.

To investigate the stability of the top-ranked three complexes, we also determined the MM-PBSA and MM-GBSA binding energies for these complexes **(Fig 7)**. From **Fig 7A**, we observed that the average binding energies obtained from the RELA *vs*. Nilotinib, RELA *vs*. Entrectinib, and RELA *vs*. Imatinib complexes were 132.44 kJ/mol, 180.87 kJ/mol, and 79.50 kJ/mol, respectively. These positively average binding energies with YASARA indicate the significant stability of these three complexes [44]. To examine the stability of those complexes with another software, we calculated MM-GBSA binding free energy (ΔG) for the MD simulation with the Desmond module of Schrödinger **(Fig 7B)**. **Fig 7B** indicates that the average ΔG of RELA *vs*. Entrectinib, RELA vs. Imatinib, and RELA *vs*. Nilotinib are -146.24 kJ/mol, -148.59 kJ/mol and -94.84 kJ/mol, respectively. Also, the results **(S1 Fig)** show that the overall binding energy for RELA *vs*. Imatinib is -35.51 kcal/mol after adding the solvation term of 27.65 kcal/mol. Moreover, RELA *vs*. Entrectinib and RELA *vs*. Nilotinib showed the average VDW energy as -39.79 kcal/mol and -36.47 kcal/mol respectively, whereas the electrostatic energy showed -65.83 and -14.43 kcal/mol, respectively. The ΔG_Total_ and solvation energy for RELA *vs*. Entrectinib is -35 and 70.67 kcal/mol. These negative average binding energies with the Desmond module indicate the significant stability of those three complexes as before [45].

**Fig 8:**
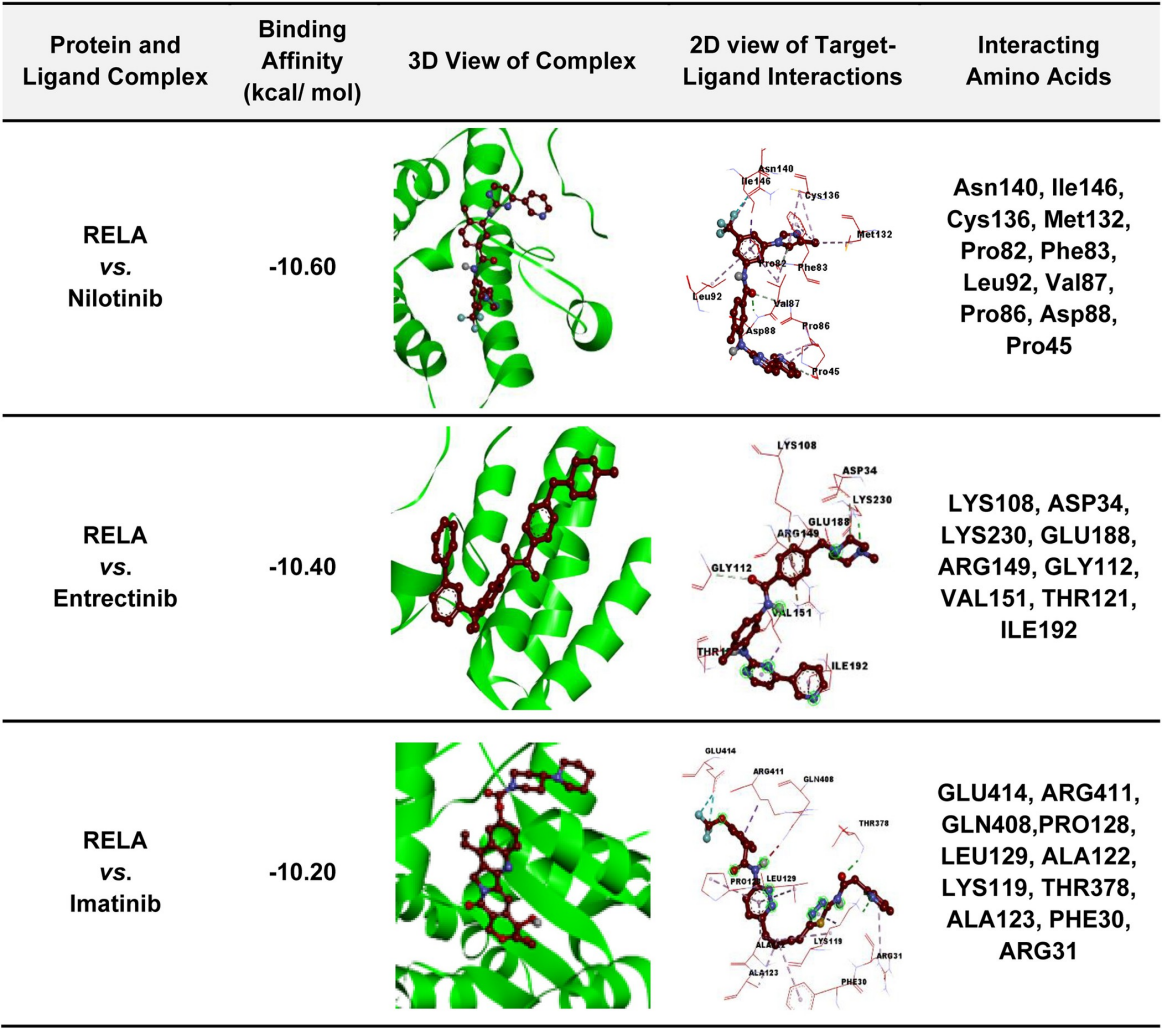
Receptor-ligand interactions (The 3D views of the highest binding affinity score-based receptor and top 3 lead compounds with their interacting amino acids).

We also investigated the drug-target binding positions in the proposed top-ranked three complexes docked by AutoDock Vina in **Fig 8**. The 2D schematic diagram of the receptor-ligand interactions is given in the fourth column. The third column displays the 3D view of the protein-ligand complex, and the interactive key amino acids in the docked complex are mentioned in the fifth column.

## 4. Discussion

Chronic lung diseases including asthma [8], tuberculosis [9], cystic fibrosis [4], pneumonia [1, 10], emphysema [11], bronchitis [12], IPF [6], COPD [5], and ILD [7] are associated with the severity of SARS-CoV-2 infections. Therefore, in this study, we identified the top-ranked 11 genes (*STAT1*, *TLR4*, *CXCL10*, *CCL2*, *JUN*, *DDX58*, *IRF7*, *ICAM1*, *MX2*, *IRF9*, and *ISG15*) as hub-sDEGs by the protein-protein interaction (PPI) network analysis of the shared differentially expressed genes (sDEGs) to disclose shared molecular mechanisms and associated drug molecules. Among the hub-sDEGs, the signal transducer and activator of transcription 1 (STAT1) is essential for the defense against viral infections like influenza A and SARS-CoV-2 infections [46]. Some authors proposed STAT1 as a therapeutic target and candidate biomarker for SARS-CoV-2 infection [47]. The toll-like receptor 4 (TLR4) plays a crucial role in the innate immune system, cardiac hypertrophy, myocardial inflammation, lung fibrosis, atherosclerosis, skin fibroblasts, and alveolar and bronchial epithelial cells that bind with the spike-glycoprotein of SARS-CoV-2 and increases angiotensin-converting enzyme 2 (ACE 2) expression. It has been suggested as a potential therapeutic target for the neurological complexities and respiratory illness of SARS-CoV-2 infections [48]. CXCL10 is a pro-inflammatory chemokine that plays a crucial role in the COVID-19-related cytokine storm and olfactory dysfunctions [49]. Moreover, it is implicated in the increased susceptibility observed among patients afflicted with severe obesity, hypertension, diabetes, and lung cancer [50]. The C-C motif ligand 2 (CCL2) is significantly involved in the pathogenesis of the breathing complexities that define the most severe form of COVID-19 [51]. The upregulation of JUN (Jun proto-oncogene, AP-1 transcription factor subunit) is prominently observed in the pathogenesis of severe COVID-19 patients with the development of viral infections and immunological abnormalities by T-cell hyperactivation [21]. The DDX58 gene also known as retinoic acid-inducible gene I (RIG-I), plays a vital role in the development progression of SARS-CoV-2 infection and regulating host immune responses including dermatomyositis (DM) characterized by muscle dysfunction, pain, skin rashes, etc. [52]. Inflammatory cytokines, including Interleukin and type I interferon, are associated with susceptibility to Dermatomyositis [52]. The gene IRF7 (Interferon Regulatory Factor 7) is associated with the pathogenesis of lung cancer, type 1 diabetes, and obesity [53]. It is also over-expressed in COVID-19, which exhibits the congruence between responses of primary lung cells and systemic blood cells during the acute phases of SARS-CoV-2 infection [54]. In COVID-19 patients, a high level of ICAM-1 (Intercellular Adhesion Molecule 1) gene is observed, induced by cytokines including Tumor Necrosis Factor-Alpha (TNF-α) and Interleukin-1 (IL-1) that regulates influenza virus infection in human bronchial epithelial cells (HBEpC) during the primary stages of infection [55]. The MX2 gene is highly enriched in the type 1 interferon signaling pathway, which plays a crucial role in the development of IPF disease and the innate immune response in the pathogenesis of COVID-19 [56]. The IRF9 is a biomarker gene for respiratory viral infection [57]. The interferon-stimulated gene 15 (ISG15) is a crucial therapeutic target for active tuberculosis and other comorbidities such as dermatomyositis, glioblastoma, psoriasis, hypertension, lung cancer, and breast cancer [58]. Thus, the association of our proposed hub-sDEGs with the progression of SARS-CoV-2 infections and different lung diseases are also supported by the literature review. The interaction network analysis of hub-sDEGs with different diseases from independent databases showed that some of our proposed hub-sDEGs (*CCL2*, *STAT1*, *MX2*, *ICAM1*, *JUN*, and *IRF7*) are significantly associated with different lung diseases including asthma, bronchiectasis, pneumonia, pulmonary fibrosis, tuberculosis, lung injury, lung neoplasms (**Fig 3** and **S3 Table**). Besides, bronchiectasis and diabetes mellitus are linked by ICAM1 and STAT1; hypertensive disease is linked by CCL2, ICAM1, and JUN; inflammation is linked by CCL2, ICAM1, and IRF7; influenza is linked by CCL2, IRF7, MX2, and STAT1; liver cirrhosis is linked by CCL2 and STAT1 and multiple sclerosis is linked by ICAM1. The genes CCL2, ICAM1, and JUN were identified as being associated with neurological disorders such as brain ischemia and schizophrenia. Schizophrenia patients are more susceptible to COVID-19 infection [59]. The genes ICAM1 and STAT1 have associations with some skin disorders such as urticaria, and eczema, respectively. Interestingly, these skin disorders have also been observed in some patients with COVID-19 [13,60]. Several studies reported that elevated risk of developing COVID-19 among patients with liver disease and rheumatoid arthritis. Moreover, these disorders were associated with increased severity of COVID-19 and lead to mortality [61,62]. Public literature also supports our findings and suggests that melanoma [63], rheumatoid arthritis [61], asthma [8], atherosclerosis [64], obesity, hypertension, diabetes mellitus [50], schizophrenia [59], multiple sclerosis and multiple myeloma [13] are closely linked to COVID-19.

To investigate the pathogenetic mechanisms of the proposed hub-sDEGs, we selected the few top common GO-terms (BPs, MFs, and CCs) from two databases (DAVID and Enrichr) and pathways that are common in at least two databases from KEGG, BioCarta, Reactome, and WikiPathways (**Tables 1** and **2**). Among the enriched BPs, defense response to virus, positive regulation of interferon-alpha/beta production, positive regulation of transcription by RNA polymerase II, positive regulation of DNA-templated transcription, and cellular response to interferon gamma are the top GO terms. Defense response to virus were reported as important BPs for SARS-CoV-2 progression and lung disease [65]. Positive regulation of interferon-alpha or beta production promotes SARS-CoV-2 pulmonary vascular infection by triggering the expression of ACE2 [66]. Positive regulation of transcription by RNA polymerase II has been identified as an important BP for COVID-19-associated cardiac dysfunctions [67]. The regulation of DNA-templated transcription is crucial for the activation of the host immune response against SARS-CoV-2 [68]. Cellular response to interferon-gamma (IFN-γ), plays a crucial role in SARS-CoV-2 infection due to its association with both inflammation and immune responses [69]. In COVID-19 patients, it is also regarded as a risk factor for pulmonary fibrosis [70]. In the enriched MFs, double-stranded DNA binding, chemokine activity [71], ubiquitin-like protein ligase binding [72], and RNA polymerase II core promoter proximal region sequence-specific DNA binding [73] are related to COVID-19. Sequence-specific double-stranded DNA binding [74] and transcription regulatory region sequence-specific DNA binding [75] are associated with the development of melanoma and ovarian cancer, respectively. According to the CCs, several significant cellular components (CCs) were identified for various diseases, the endosome membrane was found to be enriched with chronic obstructive pulmonary disease (COPD) and lung cancer [76]. Additionally, the cytoplasmic vesicle membrane has been identified as the enriched CC term, associated with the development of obesity and COVID-19 [77]. In patients with non-small-cell lung cancer (NSCLC), euchromatin has been identified as a significant CC term [78]. Furthermore, the nucleus and intracellular membrane-bounded organelle were found to be significant CC terms for COVID-19 and non-alcoholic fatty liver disease (NAFLD) [79]. Among the enriched pathways, *Coronavirus disease*, *Interferon signaling pathway*, *Immune responses*, and *Measles virus infection* are involved with the progression of SARS-CoV-2 infection and other comorbidities (**Table 2** and **S4 Table**). An essential route in the initial line of defense against SARS-CoV-2 is interferon signaling (Type I and III) [80]. COVID-19 patients have innate and adaptive immune responses and rapidly declining antibodies [81]. Measles has a significant association with acute respiratory tract infections, which can contribute to the development of acute exacerbations in individuals with chronic bronchitis, asthma, pneumonia, and COVID-19 [18].

The hub-sDEGs interaction network analysis with TFs proteins revealed that 6 TFs proteins (MYC, SOX2, CEBPA, NANOG, RELA, and MSX1) are the key transcriptional regulatory factors of hub-sDEGs (**Fig 4**). Among them, the TF-protein MYC has been demonstrated as a therapeutic target for lung cancer [28]. Activation of MYC has an association with COPD and viral infection influenza A virus (IAV) that also promotes the metabolic reprogramming of glutamine in SARS-CoV-2-infected cells [82]. Several studies demonstrated that the transcriptional activation of SOX2 is linked with tumorigenesis that leads to various cancers including glioblastoma, small cell lung cancer (SCLC), lung squamous cell carcinoma (LSCC), lung adenocarcinoma, breast cancer, and colon cancer [83]. The SARS-CoV-2 infection may be significantly impacted by the TF-protein CEBPA. The TF-protein NANOG is reported as a prognostic biomarker for lung cancer [84]. The TF-protein RELA is a regulator of both proliferative and inflammatory cellular responses, and it also plays a key role in the development of NF-kB and SARS-CoV-2 infection [85]. The TF protein MSX1 has been identified as a prognostic marker for several conditions, including colorectal cancer (CRC), breast cancer, and endometriosis [86].

To explore hub-sDEGs guided few potential repurposable therapeutic drugs for SARS-CoV-2 treatment with one or more lung diseases, at first, we performed molecular docking analysis with 184 candidate drug molecules that were collected from different published articles associated with COVID-19 and/or lung diseases, by using AutoDock Vina. We selected nine (out of 184) top-ranked drugs (Nilotinib, Entrectinib, Imatinib, SCH-772984, Icotinib, Regorafenib, Sorafenib, Ibrutinib, and Dabrafenib) that showed strong binding affinities with our proposed and independent receptor proteins **(Fig 5A and 5B, S5 and S8 Tables)**. The selected drug molecules were optimized through density functional theory (DFT) and observing their good chemical stability. To verify the significance of protein-ligand interaction by another software, we considered the well-known GLIDE module of Schrödinger. We observed that previously suggested top-ranked three molecules (Nilotinib, Entrectinib, Imatinib) significantly interact with our suggested target proteins according to the binding scores (Glide g-score, Docking score ≤ −6) and Glide e-model ≤ −35 produced by Schrödinger also (**Fig 5C**, **S9–S11 Tables**) [87]. Therefore, we considered these three molecules (Entrectinib, Imatinib, and Nilotinib) for further investigation. We evaluated the stability of the top-ranked receptor RELA with these three molecules (Entrectinib, Imatinib, and Nilotinib) by molecular dynamic simulation studies and found their stable performance **(Figs 6 and 7).** Recent research has shown that the tyrosine kinase inhibitors Entrectinib and Nilotinib both have antiviral activity that could reduce SARS-CoV-2 infections in human lung tissue [88]. Entrectinib is also recommended for the treatment of metastatic NTRK-positive solid tumors and ROS1-positive non-small cell lung cancer (NSCLC) [89]. Some studies reported that Nilotinib alone or its combination with Carboplatin and Paclitaxel could be considered as a therapy to treat a variety of cancer disorders such as ovarian cancer, colorectal cancer, and chronic myeloid leukemia [90]. Moreover, Entrectinib and Nilotinib are also considered to be potential candidate drugs for COVID-19 [56,91]. Imatinib is an FDA-approved drug used for treating chronic myeloid leukemia (CML), ovarian cancer, and gastrointestinal stromal tumors (GIST) [92]. Recent studies reported that imatinib exhibits inhibitory effects on the initial stages of SARS-CoV-2 infection, providing valuable insights into its potential as a therapeutic intervention for combating viral infection [93]. Thus, it has been found that the proposed drugs are approved by the Food and Drug Administration (FDA). Therefore, the findings of this study might be interesting resources for the diagnosis and therapies of COVID-19 patients who are also suffering from one or more lung diseases. However, experimental validation in wet-lab is required for taking a proper treatment plan.

## 5. Conclusion

In this study, we identified the top-ranked 11 genes (*STAT1*, *TLR4*, *CXCL10*, *CCL2*, *JUN*, *DDX58*, *IRF7*, *ICAM1*, *MX2*, *IRF9*, and *ISG15*) as the hub of the shared differentially expressed genes (hub-sDEGs) highlighting their molecular mechanisms for which SARS-CoV-2 infections and different lung diseases stimulate each other. The GO enrichment analysis with hub-sDEGs revealed that these hub genes are involved in cellular response to interferon-gamma, defense response to virus, positive regulation of interferon-alpha or beta production, etc., and pathways were enriched in coronavirus disease, TNF signaling pathway, interferon signaling pathway, etc., that are associated with both COVID-19 and lung diseases. Some TFs proteins (MYC, SOX2, CEBPA, NANOG, RELA, and MSX1) and miRNAs (hsa-miR-16-5p, hsa-miR-129-2-3p, hsa-miR-21-3p, hsa-miR-27a-5p, hsa-miR-1-3p, and hsa-miR-155-5p) were also detected as the key transcriptional and post-transcriptional regulatory factors of key/hub-sDEGs. Then, hub-sDEGs guided top-ranked three repurposable drug molecules (Entrectinib, Imatinib, and Nilotinib) were detected by molecular docking analysis with AutoDock Vina and GLIDE module of Schrödinger. Additionally, molecular dynamic simulations confirmed the stability of the top-ranked three drugs (Entrectinib, Imatinib, and Nilotinib) in complex with the RELA protein over a 100 ns period. Therefore, the findings of this study would be useful resources for the diagnosis and therapies of COVID-19 patients who are also suffering from chronic lung diseases.

## Supporting information

S1 Table Descriptions of gene expression datasets with their geo features. (DOCX)

S2 Table List of 26 review articles with their identified drug molecules. (DOCX)

S3 Table List of diseases that are associated with 6 hub-sDEGs among our proposed 11 hub-sDEGs based on the DisGeNET database. (DOCX)

S4 Table Pathway enrichment analysis of hub-sDEGs based on KEGG, BioCarta, Reactome, and WikiPathways databases. (DOCX)

S5 Table Binding affinity score (BAS) of the top 30 drugs among the 184 drugs against the proposed 17 drug target proteins obtained by AutoDock Vina through molecular docking analysis. (XLSX)

S6 Table List of 27 published articles associated with SARS-CoV-2 infections with their identified hub-genes. (DOCX)

S7 Table List of 27 published articles associated with lung disease with their identified hub-genes. (DOCX)

S8 Table Binding affinity score (BAS) of the top-ranked 30 (out of 184) published drugs against the published top-ranked 8 independent meta receptors for cross-validation. (XLSX)

S9 Table Glide g-score of the top nine drugs among the 184 drugs against the proposed 17 drug target proteins obtained by the GLIDE module of Schrödinger. (XLSX)

S10 Table Docking score of the top nine drugs among the 184 drugs against the proposed 17 drug target proteins obtained by the GLIDE module of Schrödinger. (XLSX)

S11 Table Glide e-model score of the top nine drugs among the 184 drugs against the proposed 17 drug target proteins obtained by the GLIDE module of Schrödinger. (XLSX)

S1 Fig The representation of binding energy of the top-ranked receptor RELA with the top-ranked three molecules (Entrectinib, Imatinib, and Nilotinib) was calculated using the MM-GBSA approach from MD simulation trajectories. (TIF)

## Abbreviations

GEO: Gene Expression Omnibus
COPD: Chronic Obstructive Pulmonary Disease
IPF: Idiopathic Pulmonary Fibrosis
ILD: Interstitial Lung Disease
CF: Cystic Fibrosis
DEGs: Differentially Expressed Genes
sDEGs: Shared Differentially Expressed Genes
PPI: Protein–Protein Interaction
GO: Gene Ontology
KEGG: Kyoto Encyclopedia of Genes and Genomes
TFs: Transcription Factors
miRNAs: Micro-RNAs
PDB: Protein Data Bank
WHO: World Health Organization
DFT: Density Functional Theory
VDW: Van Der Waals
