# Supplementary material for: In-silico discovery of common molecular signatures for which SARS-CoV-2 infections and lung diseases stimulate each other, and drug repurposing
Source: PLoS One. 2024 Jul 18;19(7):e0304425. doi: 10.1371/journal.pone.0304425 (PMC11257407; doi:10.1371/journal.pone.0304425)
Supplement: S1 Table — (DOCX) [file pone.0304425.s001.docx]

**S1 Table.** Descriptions of gene expression datasets with their geo features.

| **Disease Name** | **Data Type** | **NCBI-Accession ID** | **Countries of data collection** | **GEO Platform** | **Array Type/ Platform** | **No. of Genes** | **No. of patients**  **(Case/control)** |
| --- | --- | --- | --- | --- | --- | --- | --- |
| SARS-CoV-2 [1-3] | RNA-Seq | GSE147507 | USA | GPL18573 | Illumina NextSeq 500 | 21797 | 18/29 |
|  |  | GSE150392 | USA |  |  | 36942 | 3/3 |
|  |  | GSE152075 | USA |  |  | 35784 | 430/54 |
| COPD [4] | Microarray | GSE100281 | United Kingdom | GPL11532 | Affymetrix Human Gene 1.1 ST Array | 18625 | 79/16 |
| IPF [5] |  | GSE53845 | USA | GPL6480 | Agilent-014850 Whole Human Genome Microarray 4x44K G4112F | 41000 | 40/8 |
| ILD [6] |  | GSE40839 | United Kingdom | GPL96 | Affymetrix Human Genome U133A Array | 22215 | 11/10 |
| Asthma [7] |  | GSE64913 | United Kingdom | GPL570 | Affymetrix Human Genome U133 Plus 2.0 Array | 54675 | 28/42 |
| Tuberculosis [8] |  | GSE34608 | Germany | GPL6480 | Agilent-014850 Whole Human Genome Microarray 4x44K G4112F | 32839 | 8/18 |
| Cystic Fibrosis [9] |  | GSE107846 | USA | GPL13369 | Illumina Human Whole-Genome DASL HT | 47323 | 28/12 |
| Pneumonia [10] |  | GSE42830 | United Kingdom | GPL10558 | Illumina HumanHT-12 V4.0 expression beadchip | 47323 | 8/38 |
| Emphysema [11] |  | GSE1122 | USA | GPL80 | Affymetrix Human Full Length HuGeneFL Array | 7129 | 10/5 |
| Bronchitis [12] |  | GSE22148 | United Kingdom | GPL570 | Affymetrix Human Genome U133 Plus 2.0 Array | 54675 | 72/71 |

**References**

1. Blanco-Melo D, Nilsson-Payant BE, Liu W-C, Uhl S, Hoagland D, Møller R, et al. Imbalanced host response to SARS-CoV-2 drives development of COVID-19. Cell. 2020;181(5):1036-45. e9.

2. Sharma A, Garcia Jr G, Wang Y, Plummer JT, Morizono K, Arumugaswami V, et al. Human iPSC-derived cardiomyocytes are susceptible to SARS-CoV-2 infection. Cell Reports Medicine. 2020;1(4):100052.

3. Lieberman NA, Peddu V, Xie H, Shrestha L, Huang M-L, Mears MC, et al. In vivo antiviral host transcriptional response to SARS-CoV-2 by viral load, sex, and age. PLoS biology. 2020;18(9):e3000849.

4. Willis-Owen SA, Thompson A, Kemp PR, Polkey MI, Cookson WO, Moffatt MF, et al. COPD is accompanied by co-ordinated transcriptional perturbation in the quadriceps affecting the mitochondria and extracellular matrix. Scientific reports. 2018;8(1):1-9.

5. DePianto DJ, Chandriani S, Abbas AR, Jia G, N'Diaye EN, Caplazi P, et al. Heterogeneous gene expression signatures correspond to distinct lung pathologies and biomarkers of disease severity in idiopathic pulmonary fibrosis. Thorax. 2015;70(1):48-56.

6. Lindahl GE, Stock CJ, Shi-Wen X, Leoni P, Sestini P, Howat SL, et al. Microarray profiling reveals suppressed interferon stimulated gene program in fibroblasts from scleroderma-associated interstitial lung disease. Respiratory research. 2013;14(1):1-14.

7. Singhania A, Rupani H, Jayasekera N, Lumb S, Hales P, Gozzard N, et al. Altered epithelial gene expression in peripheral airways of severe asthma. PLoS One. 2017;12(1):e0168680.

8. Maertzdorf J, Weiner 3rd J, Mollenkopf H-J, Network T, Bauer T, Prasse A, et al. Common patterns and disease-related signatures in tuberculosis and sarcoidosis. Proceedings of the National Academy of Sciences. 2012;109(20):7853-8.

9. Kopp BT, Thompson R, Kim J, Konstan R, Diaz A, Smith B, et al. Secondhand smoke alters arachidonic acid metabolism and inflammation in infants and children with cystic fibrosis. Thorax. 2019;74(3):237-46.

10. Bloom CI, Graham CM, Berry MP, Rozakeas F, Redford PS, Wang Y, et al. Transcriptional blood signatures distinguish pulmonary tuberculosis, pulmonary sarcoidosis, pneumonias and lung cancers. PloS one. 2013;8(8):e70630.

11. Golpon HA, Coldren CD, Zamora MR, Cosgrove GP, Moore MD, Tuder RM, et al. Emphysema lung tissue gene expression profiling. American journal of respiratory cell and molecular biology. 2004;31(6):595-600.

12. Singh D, Fox SM, Tal-Singer R, Plumb J, Bates S, Broad P, et al. Induced sputum genes associated with spirometric and radiological disease severity in COPD ex-smokers. Thorax. 2011;66(6):489-95.
