# Supplementary material for: In-silico discovery of common molecular signatures for which SARS-CoV-2 infections and lung diseases stimulate each other, and drug repurposing
Source: PLoS One. 2024 Jul 18;19(7):e0304425. doi: 10.1371/journal.pone.0304425 (PMC11257407; doi:10.1371/journal.pone.0304425)
Supplement: S2 Table — (DOCX) [file pone.0304425.s002.docx]

**S2 Table.** List of 26 review articles with their identified drug molecules.

| **Literature** | **Proposed Drugs** | **Common drugs with at least four literatures** | **Common drugs with at least three literatures** | **Common drugs with at least two literatures** |
| --- | --- | --- | --- | --- |
| [1] | Fostamatinib, Linagliptin, Lysergol and Sophoridine | Chloroquine,  Hydroxychloroquine, Remdesivir, Ritonavir, Ivermectin, Favipiravir, Dexamethasone, Nitazoxanide, Azithromycin, Camostat, Baricitinib, Lopinavir, Tocilizumab, Ritonavir, | Gefitinib, Dasatinib, Favipiravir, Azithromycin, Chloroquine,  Hydroxychloroquine, Remdesivir, Lopinavir, Ritonavir, Ivermectin, Favipiravir, Dexamethasone, Nitazoxanide, Azithromycin, Camostat, Methylprednisolone, Baricitinib, Daclatasvir, Darunavir, Nafamostat, Tocilizumab, Imatinib, Tacrolimus | Rapamycin, Tacrolimus, Ivermectin, Daclatasvir,  Vemurafenib, Erlotinib, Bosutinib, Imatinib, Ponatinib, Regorafenib, Vandetanib, Entrectinib, Curcumin, Tamoxifen, Tocilizumab, Gemcitabine, Gefitinib, Dasatinib, Favipiravir, Azithromycin, Chloroquine,  Hydroxychloroquine, Remdesivir, Lopinavir, Ritonavir, Fostamatinib, Colchicine, Ivermectin, Favipiravir, Dexamethasone, Nitazoxanide, Azithromycin, Camostat, Methylprednisolone, Baricitinib, Sofosbuvir, Darunavir, Danoprevir, Meplazumab, Nafamostat, Sarilumab, Umifenovir, Bamlanivimab, Ruxolitinib, Eculizumab, Hydrocortisone, Testosterone, Molnupiravir, Nirmatrelvir, Ritonavir, Vilobelimab, Bevacizumab, Vilobelimab, Melatonin, Captopril, Mefloquine, Homoharringtonine, Ribavirin, Boceprevir, Sorafenib, Obatoclax, Sulfasalazine,  Nintedanib |
| [2] | Chloroquine, Hydroxychloroquine, Ivermectin, Favipiravir, Colchicine, Remdesivir, Dexamethasone, Nitazoxanide, Azithromycin, Camostat, Methylprednisolone, Baricitinib |  |  |  |
| [3] | Remdesivir, Favipiravir, Sofosbuvir, Daclatasvir, Lopinavir, Darunavir, Danoprevir, Meplazumab, Nafamostat, Camostat, Triazavirin, Umifenovir, Bamlanivimab, Slidenafil, Tadalafil, Finasteride Dutasteride, Dexamethasone, Tocilizumab Sarilumab, Paracetamol Ibuprofen, Ruxolitinib, Baricitinib, Eculizumab |  |  |  |
| [4] | Allopurinol, Methsuximide, Beta Carotene, Latanoprost, Famotidine, Alprostadil, Azelaic Acid, Oxytetracycline, Hydrocortisone, Enzalutamide, Aminopterin, Tafenoquine, Arformoterol, Novobiocin, Lidocaine, Apalutamide, Bupivacaine, Propranolol, Baloxavir Marboxil, Estradiol Acetate, Testosterone, Lovastatin, Prednisone, Formoterol, Brinzolamide, Testosterone Cypionate, Azathioprine, Cannabidivarin, Cefamandole. |  |  |  |
| [5] | Remdesivir, Molnupiravir, Nirmatrelvir, Ritonavir, Ensitrelvir, Bebtelovimab, Regdanvimab, Sotrovimab, Amubarvimab, Romlusevimab, Bamlanivimab, Etesevimab, Casirivimab, Imdevimab, Cilgavimab, Tixagevimab, Baricitinib, Tofacitinib, Tocilizumab, Sarilumab, Anakinra, Vilobelimab |  |  |  |
| [6] | Colchicine, Ramipril, Methylprednisolone, Fingolimod, Tranilast, DC-SIGN, Baricitinib, BDB-001, Bevacizumab, Eculizumab, Meplazumab, Tocilizumab (TCZ), Vilobelimab, MART-10, Melatonin, Toradol, APN01, Captopril, Camostat mesylate, Nafamostat mesylate, Arbidol, Azithromycin, Cepharanthine, Selamectin, Mefloquine hydrochloride, Chloroquine, Darunavir, Emetine, Favipiravir, Homoharringtonine, Hydroxychloroquine, Ivermectin, Lopinavir, Remdesivir, Ribavirin, Ritonavir |  |  |  |
| [7] | Boceprevir, Lonafarnib, Nirmatrelvir, Nelfinavir, Molnupiravir, Apilimod, Remdesivir, Niclosamide, Lactoferrin, Nafamostat, Proguanil, Omipalisib, Sorafenib, Obatoclax, Imatinib, Cyclosporine, Otamixaban, Sulfasalazine, Dexamethasone, Baricitinib, Tacrolimus, Zotatifin, Nintedanib, Melatonin, Carvedilol |  |  |  |
| [8] | Rapamycin, Tacrolimus, Torin-2, Redotinib, Ivermectin, Danoprevir, Daclatasvir |  |  |  |
| [9] | Phorbol 12-Myristate 13-Acetate (PMA) (S7791), Trametinib (S2673), Selumetinib (S1008), SCH-772984 (S7101), Vemurafenib (S1267), Gefitinib (S1025) And Erlotinib (S7786), LPS (L2630), Dexamethasone (D4902) and Hydrocortisone (3867) |  |  |  |
| [10] | Apoptone, Dexibuprofen, Docetaxel, Eribulin, Ibuprofen, Isosorbide, Navitoclax, Obatoclax, Paclitaxel, Paclitaxel Docosahexaenoic Acid, Rasagiline and Venetoclax, Bosutinib, Brigatinib, Dasatinib, Imatinib, Nilotinib, Ponatinib, Regorafenib, Dabrafenib, Encorafenib, Ripretinib, Sorafenib, Vemurafenib, Afatinib, Dacomitinib, Erlotinib, Gefitinib, Icotinib, Lapatinib, Neratinib, Olmutinib, Osimertinib, Vandetanib, Zanubrutinib, Entrectinib, Fedratinib, Ruxolitinib, Everolimus, Ridaforolimus, Temsirolimus, Midostaurin. |  |  |  |
| [11] | Astragalin, 4-P-Coumaroylquinic Acid, 3-P-Coumaroylquinic Acid, Sinapoyl-D-Glucoside, 1- Sinapoyl-D- Glucose. |  |  |  |
| [12] | Glycine and Pyridoxal Phosphate |  |  |  |
| [13] | Suloctidil HL60 UP, Prenylamine HL60 UP, Acetohexamide PC3 UP, Chlorophyllin CTD 00000324, 3’-Azido-3’-Deoxythymidine CTD 00007047, Prochlorperazine MCF7 UP, Terfenadine HL60 UP, Etoposide HL60 UP, Arsenenous Acid, CTD 00000922, Propofol MCF7 UP. |  |  |  |
| [14] | Torin2, Rapamycin, Radotinib, Ivermectin, Thiostrepton, Tacrolimus, Daclatasvir |  |  |  |
| [15] | Auranofin, Azelastine, Digoxin, Vinblastine, Fluvastatin, And Methodextrate |  |  |  |
| [16] | Fostamatinib, Entrectinib, Ponatinib, Dasatinib, Bosutinib, Ibrutinib, Acalabrutinib, Resveratrol, Nintedanib, Vandetanib, Gefitinib, Sulfasalazine, Mesalamine, Curcumin, Rilonacept, Alteplase, Aspirin, Bevacizumab, Omeprazole, Naproxen, Cisplatin |  |  |  |
| [17] | Rutin, NADH, Ginsenoside Rg1, Protopanaxatriol |  |  |  |
| [18] | Naproxol, Teniposide, Amsacrine, BRD-K68548958, Palbociclib |  |  |  |
| [19] | Chloroquine, Remdesivir, Favipiravir, Curcumin, Chromium, Ellipticine, Tamoxifen, and Deguelin. |  |  |  |
| [20] | MIGLITOL, Bosentan, Coenzyme Q10, Metoprolol HL60 UP, Chelidonine HL60 DOWN, Sildenafil, Norepinephrine, Dydrogesterone and 1,3-Dimethylthiourea |  |  |  |
| [21] | Remdesivir, Chloroquine/Hydroxychloroquine, Azithromycin, Lopinavir/Ritonavir, Tocilizumab, Methylprednisolone (Steroids), Convalescent Plasma, Umifenovir (Arbidol), Favipiravir/Favilavir, Sofosbuvir, Baricitinib, Camostat, Emapalumab, Anakinra |  |  |  |
| [22] | Lopinavir/Ritonavir, Remdesivir, Hydroxychloroquine, Steroids, Tocilizumab, Azithromycin |  |  |  |
| [23] | Hydroxychloroquine, Chloroquine, Lopinavir, Remdesivir, Favipiravir, Ribavirin, Azithromycin, Ritonavir (RTV), Darnunavir |  |  |  |
| [24] | Amodiaquine, Captopril, Chloroquine, Cyclosporin A, Chlorpromazine Hydrochloride, Clomipramine, Disulfiram, Enalapril, Gemcitabine Hydrochloride, Hydroxychloroquine, Dasatinib, Imatinib Mesylate, Loperamide, Mefloquine, Metformin, Nitazoxanide, Promethazine Hydrochloride, Remdesivir, Tamoxifen, Terconazole, Toremifene, Teicoplanin |  |  |  |
| [25] | Umbelliprenin, Quercetin, Kaempferol, Luteolin, Praeruptorin E, Stigmasterol, and Oroxylin A. |  |  |  |
| [26] | Vismodegib, Gemcitabine, Clofazimine, Celecoxib, Brequinar, Conivaptan, Bedaquiline and Tolcapone, Homoharringtonine, Salinomycin, Boceprevir, Tilorone and Chloroquine, Darunavir, Ritonavir, and Lopinavir |  |  |  |

**References**

1. Yao Y, Zhang Y, Li Z, Chen Z, Wang X, Li Z, et al. A deep learning-based drug repurposing screening and validation for anti-SARS-CoV-2 compounds by targeting the cell entry mechanism. bioRxiv. 2023:2023.06. 03.543589.

2. Vaz ES, Vassiliades SV, Giarolla J, Polli MC, Parise-Filho R. Drug repositioning in the COVID-19 pandemic: fundamentals, synthetic routes, and overview of clinical studies. European Journal of Clinical Pharmacology. 2023:1-29.

3. Kumar S, Basu M, Ghosh P, Pal U, Ghosh MK. COVID-19 therapeutics: Clinical application of repurposed drugs and futuristic strategies for target-based drug discovery. Genes & Diseases. 2023.

4. Wang X, Wang H, Yin G, Zhang YD. Network-based drug repurposing for the treatment of COVID-19 patients in different clinical stages. Heliyon. 2023;9(3).

5. Li G, Hilgenfeld R, Whitley R, De Clercq E. Therapeutic strategies for COVID-19: progress and lessons learned. Nature Reviews Drug Discovery. 2023:1-27.

6. Raghav PK, Mann Z, Ahluwalia SK, Rajalingam R. Potential treatments of COVID-19: Drug repurposing and therapeutic interventions. Journal of Pharmacological Sciences. 2023;152(1):1-21.

7. Wang R-S, Loscalzo J. Repurposing drugs for the treatment of COVID-19 and its cardiovascular manifestations. Circulation Research. 2023;132(10):1374-86.

8. Ahmed FF, Reza MS, Sarker MS, Islam MS, Mosharaf MP, Hasan S, et al. Identification of host transcriptome-guided repurposable drugs for SARS-CoV-1 infections and their validation with SARS-CoV-2 infections by using the integrated bioinformatics approaches. PloS one. 2022;17(4):e0266124.

9. Sanchez-Burgos L, Gómez-López G, Al-Shahrour F, Fernandez-Capetillo O. An in silico analysis identifies drugs potentially modulating the cytokine storm triggered by SARS-CoV-2 infection. Scientific reports. 2022;12(1):1-10.

10. Siminea N, Popescu V, Sanchez Martin JA, Florea D, Gavril G, Gheorghe A-M, et al. Network analytics for drug repurposing in COVID-19. Briefings in bioinformatics. 2022;23(1):bbab490.

11. Jose S, Gupta M, Sharma U, Quintero-Saumeth J, Dwivedi M. Potential of phytocompounds from Brassica oleracea targeting S2-domain of SARS-CoV-2 spike glycoproteins: Structural and molecular insights. Journal of molecular structure. 2022;1254:132369.

12. Hasan MI, Rahman MH, Islam MB, Islam MZ, Hossain MA, Moni MA. Systems Biology and Bioinformatics approach to Identify blood based signatures molecules and drug targets of patient with COVID-19. Informatics in Medicine Unlocked. 2022;28:100840.

13. Chen Q, Xia S, Sui H, Shi X, Huang B, Wang T. Identification of hub genes associated with COVID-19 and idiopathic pulmonary fibrosis by integrated bioinformatics analysis. PloS one. 2022;17(1):e0262737.

14. Mosharaf M, Reza M, Kibria M, Ahmed FF, Kabir M, Hasan S, et al. Computational identification of host genomic biomarkers highlighting their functions, pathways and regulators that influence SARS-CoV-2 infections and drug repurposing. Scientific reports. 2022;12(1):1-22.

15. Turilli ES, Lualdi M, Fasano M. Looking at COVID-19 from a Systems Biology Perspective. Biomolecules. 2022;12(2):188.

16. El-Aarag SA, Mahmoud A, ElHefnawi M. Identifying potential novel insights for COVID-19 pathogenesis and therapeutics using an integrated bioinformatics analysis of host transcriptome. International journal of biological macromolecules. 2022;194:770-80.

17. Alanazi KM, Farah MA, Hor Y-Y. Multi-Targeted Approaches and Drug Repurposing Reveal Possible SARS-CoV-2 Inhibitors. Vaccines. 2021;10(1):24.

18. Auwul MR, Rahman MR, Gov E, Shahjaman M, Moni MA. Bioinformatics and machine learning approach identifies potential drug targets and pathways in COVID-19. Briefings in bioinformatics. 2021;22(5):bbab120.

19. Mahmud SH, Al-Mustanjid M, Akter F, Rahman MS, Ahmed K, Rahman MH, et al. Bioinformatics and system biology approach to identify the influences of SARS-CoV-2 infections to idiopathic pulmonary fibrosis and chronic obstructive pulmonary disease patients. Briefings in Bioinformatics. 2021;22(5):bbab115.

20. Taz TA, Ahmed K, Paul BK, Al-Zahrani FA, Mahmud SH, Moni MA. Identification of biomarkers and pathways for the SARS-CoV-2 infections that make complexities in pulmonary arterial hypertension patients. Briefings in Bioinformatics. 2021;22(2):1451-65.

21. Boettler T, Newsome PN, Mondelli MU, Maticic M, Cordero E, Cornberg M, et al. Care of patients with liver disease during the COVID-19 pandemic: EASL-ESCMID position paper. JHEP reports. 2020;2(3):100113.

22. Venturini E, Montagnani C, Garazzino S, Donà D, Pierantoni L, Lo Vecchio A, et al. Treatment of children with COVID-19: position paper of the Italian Society of Pediatric Infectious Disease. Italian Journal of Pediatrics. 2020;46(1):1-11.

23. Barcelo D. An environmental and health perspective for COVID-19 outbreak: meteorology and air quality influence, sewage epidemiology indicator, hospitals disinfection, drug therapies and recommendations. Journal of Environmental Chemical Engineering. 2020;8(4):104006.

24. Serafin MB, Bottega A, Foletto VS, da Rosa TF, Hörner A, Hörner R. Drug repositioning is an alternative for the treatment of coronavirus COVID-19. International journal of antimicrobial agents. 2020;55(6):105969.

25. Huang Y, Zheng W-j, Ni Y-s, Li M-s, Chen J-k, Liu X-h, et al. Therapeutic mechanism of Toujie Quwen granules in COVID-19 based on network pharmacology. BioData mining. 2020;13(1):1-21.

26. Lalmuanawma S, Hussain J, Chhakchhuak L. Applications of machine learning and artificial intelligence for Covid-19 (SARS-CoV-2) pandemic: A review. Chaos, Solitons & Fractals. 2020;139:110059.
