# Supplementary material for: In-silico discovery of common molecular signatures for which SARS-CoV-2 infections and lung diseases stimulate each other, and drug repurposing
Source: PLoS One. 2024 Jul 18;19(7):e0304425. doi: 10.1371/journal.pone.0304425 (PMC11257407; doi:10.1371/journal.pone.0304425)
Supplement: S3 Table — (DOCX) [file pone.0304425.s003.docx]

**S3 Table.** List of diseases that are associated with 6 hub-sDEGs among our proposed 11 hub-sDEGs based on the DisGeNET database.

| Gene | Disease | Gene | Disease |
| --- | --- | --- | --- |
| ICAM1 | Asthma | CCL2 | Manic |
| ICAM1 | Atherosclerosis | CCL2 | Metabolic Syndrome X |
| ICAM1 | Behcet Syndrome | CCL2 | Bipolar I disorder |
| ICAM1 | Bronchiectasis | CCL2 | Mycobacterium Tuberculosis, Susceptibility To (Finding) |
| ICAM1 | Cardiovascular Diseases | CCL2 | Coronary Artery Disease |
| ICAM1 | Brain Ischemia | CCL2 | Chemical and Drug Induced Liver Injury |
| ICAM1 | Cholestasis | CCL2 | Spina Bifida |
| ICAM1 | Ulcerative Colitis | CCL2 | Hepatitis, Autoimmune |
| ICAM1 | Colonic Neoplasms | IRF7 | Inflammation |
| ICAM1 | Diabetes Mellitus, Experimental | IRF7 | Immunologic Deficiency Syndromes |
| ICAM1 | Diabetes Mellitus, Non-Insulin-Dependent | IRF7 | Influenza |
| ICAM1 | Diabetic Retinopathy | IRF7 | Unipolar Depression |
| ICAM1 | Diaphragmatic Hernia | IRF7 | Major Depressive Disorder |
| ICAM1 | Hypercholesterolemia | IRF7 | Autosomal recessive predisposition |
| ICAM1 | Hypertensive disease | IRF7 | Immunodeficiency 39 |
| ICAM1 | Inflammation | JUN | Brain Ischemia |
| ICAM1 | Malaria | JUN | Colonic Neoplasms |
| ICAM1 | Mouth Neoplasms | JUN | Hypertensive disease |
| ICAM1 | Multiple Sclerosis | JUN | Peripheral Neuropathy |
| ICAM1 | Myocardial Infarction | JUN | Reperfusion Injury |
| ICAM1 | Myocardial Reperfusion Injury | JUN | Schizophrenia |
| ICAM1 | Nephrosis | JUN | Neoplastic Cell Transformation |
| ICAM1 | Obesity | JUN | Heat Stroke |
| ICAM1 | Peripheral Neuropathy | JUN | Hemangiosarcoma |
| ICAM1 | Pleurisy | JUN | Intestinal Polyps |
| ICAM1 | Prostatic Neoplasms | JUN | Liver neoplasms |
| ICAM1 | Reperfusion Injury | JUN | Lung Neoplasms |
| ICAM1 | Respiratory Syncytial Virus Infections | JUN | Osteosarcoma |
| ICAM1 | Retinal Diseases | JUN | Status Epilepticus |
| ICAM1 | Schizophrenia | JUN | Stomach Neoplasms |
| ICAM1 | Shock, Hemorrhagic | JUN | Mercury Poisoning, Nervous System |
| ICAM1 | Cerebrovascular accident | JUN | Mammary Neoplasms |
| ICAM1 | Uremia | JUN | Liver carcinoma |
| ICAM1 | Urticaria | JUN | Juvenile arthritis |
| ICAM1 | Bronchial Hyperreactivity | MX2 | Influenza |
| ICAM1 | Myocardial Ischemia | MX2 | melanoma |
| ICAM1 | Acute Lung Injury | STAT1 | Bronchiectasis |
| ICAM1 | Leukostasis | STAT1 | Immunologic Deficiency Syndromes |
| ICAM1 | Infarction, Middle Cerebral Artery | STAT1 | Influenza |
| ICAM1 | Plasmodium Falciparum Blood Infection Level | STAT1 | Autosomal recessive predisposition |
| CCL2 | Asthma | STAT1 | Liver carcinoma |
| CCL2 | Atherosclerosis | STAT1 | Liver Cirrhosis |
| CCL2 | Cardiovascular Diseases | STAT1 | Autoimmune hemolytic anemia |
| CCL2 | Brain Ischemia | STAT1 | Rheumatoid Arthritis |
| CCL2 | Hypertensive disease | STAT1 | Candidiasis, Chronic Mucocutaneous |
| CCL2 | Inflammation | STAT1 | Cytomegalovirus Infections |
| CCL2 | Mouth Neoplasms | STAT1 | Diabetes Mellitus, Insulin-Dependent |
| CCL2 | Myocardial Reperfusion Injury | STAT1 | Diarrhea |
| CCL2 | Reperfusion Injury | STAT1 | Eczema |
| CCL2 | Schizophrenia | STAT1 | Enterocolitis |
| CCL2 | Bronchial Hyperreactivity | STAT1 | Hepatosplenomegaly |
| CCL2 | Myocardial Ischemia | STAT1 | Hypertension, Renovascular |
| CCL2 | Infarction, Middle Cerebral Artery | STAT1 | Lymphopenia |
| CCL2 | Influenza | STAT1 | Mycobacterium Infections |
| CCL2 | Unipolar Depression | STAT1 | Mycobacterium Infections, Nontuberculous |
| CCL2 | Major Depressive Disorder | STAT1 | Osteopenia |
| CCL2 | Status Epilepticus | STAT1 | Osteoporosis |
| CCL2 | Bipolar Disorder | STAT1 | Delayed Puberty |
| CCL2 | Calcinosis | STAT1 | Renal Artery Stenosis |
| CCL2 | Squamous cell carcinoma | STAT1 | Thyroiditis |
| CCL2 | Carotid Artery Diseases | STAT1 | Villous atrophy of intestine |
| CCL2 | Crohn Disease | STAT1 | Herpes encephalitis |
| CCL2 | Diabetic Nephropathy | STAT1 | Carotid artery aneurysm |
| CCL2 | Muscular Dystrophy, Duchenne | STAT1 | Short stature |
| CCL2 | Endometrial Neoplasms | STAT1 | Delayed bone age |
| CCL2 | Glomerulonephritis | STAT1 | Villous atrophy |
| CCL2 | Heart valve disease | STAT1 | Recurrent upper respiratory tract infection |
| CCL2 | HIV Infections | STAT1 | Acquired hypothyroidism |
| CCL2 | Hyperalgesia | STAT1 | Generalized osteopenia |
| CCL2 | Hyperoxaluria | STAT1 | Brain Aneurysm |
| CCL2 | Hypersensitivity | STAT1 | Arthritis, Experimental |
| CCL2 | Pulmonary Hypertension | STAT1 | Cerebral arterial aneurysm |
| CCL2 | Kidney Failure, Chronic | STAT1 | hearing impairment |
| CCL2 | Premature Obstetric Labor | STAT1 | Small intestine biopsy shows villous atrophy |
| CCL2 | Leishmaniasis, Cutaneous | STAT1 | Phenotypic variability |
| CCL2 | Liver Cirrhosis | STAT1 | Highly variable clinical phenotype |
| CCL2 | Liver Cirrhosis, Experimental | STAT1 | Immune dysregulation |
| CCL2 | Liver diseases | STAT1 | Highly variable phenotype and severity |
| CCL2 | Multiple Myeloma | STAT1 | Low B cell count |
| CCL2 | Neoplasm Invasiveness | STAT1 | Variable degree of villous atrophy |
| CCL2 | Nephritis, Interstitial | STAT1 | Highly variable phenotype, even within families |
| CCL2 | Neural Tube Defects | STAT1 | Duodenal villous atrophy |
| CCL2 | Pleural Diseases | STAT1 | Immunodeficiency 31B |
| CCL2 | Pneumonia | STAT1 | Candidiasis, Familial, 7 |
| CCL2 | Pulmonary Fibrosis | STAT1 | Generalized osteoporosis with pathologic fractures |
| CCL2 | Respiratory Distress Syndrome, Adult | STAT1 | Recurrent respiratory infections |
| CCL2 | Retinal Degeneration | STAT1 | Poor bladder function |
| CCL2 | Silicosis | STAT1 | Immunodeficiency 31A |
| CCL2 | Compression of spinal cord | STAT1 | Biopsy shows villous atrophy |
| CCL2 | Thyroid Diseases | STAT1 | Large artery calcification |
| CCL2 | Thyroid Neoplasm | STAT1 | Recurrent mycobacterial infections |
| CCL2 | Tuberculosis, Pulmonary | STAT1 | Susceptibility to herpesvirus |
| CCL2 | Lung Injury |  |  |
