# Supplementary material for: In-silico discovery of common molecular signatures for which SARS-CoV-2 infections and lung diseases stimulate each other, and drug repurposing
Source: PLoS One. 2024 Jul 18;19(7):e0304425. doi: 10.1371/journal.pone.0304425 (PMC11257407; doi:10.1371/journal.pone.0304425)
Supplement: S4 Table — (DOCX) [file pone.0304425.s004.docx]

**S4 Table.** Pathway enrichment analysis of hub-sDEGs based on KEGG, BioCarta, Reactome, and WikiPathways databases.

| **Database** | **Databases Pathways** | **Adjusted *p*-value** | **Associated hub-sDEGs** |
| --- | --- | --- | --- |
| KEGG | Influenza A | 9.82E-16 | CXCL10; DDX58; STAT1; MX2; IRF7; CCL2; TLR4; IRF9; ICAM1 |
|  | Coronavirus disease | 7.63E-15 | CXCL10; JUN; DDX58; STAT1; MX2; CCL2; ISG15; TLR4; IRF9 |
|  | Epstein-Barr virus infection | 4.39E-13 | CXCL10; JUN; DDX58; STAT1; IRF7; ISG15; IRF9; ICAM1 |
|  | Measles | 4.71E-12 | JUN; DDX58; STAT1; MX2; IRF7; TLR4; IRF9 |
|  | Hepatitis C | 1.65E-09 | CXCL10; DDX58; STAT1; MX2; IRF7; IRF9 |
|  | NOD-like receptor signaling pathway | 3.26E-09 | JUN; STAT1; IRF7; CCL2; TLR4; IRF9 |
|  | toll-like receptor signaling pathway | 1.93E-08 | CXCL10; JUN; STAT1; IRF7; TLR4 |
|  | Hepatitis B | 1.58E-07 | JUN; DDX58; STAT1; IRF7; TLR4 |
|  | Kaposi sarcoma-associated herpesvirus infection | 3.38E-07 | JUN; STAT1; IRF7; IRF9; ICAM1 |
|  | RIG-I-like receptor signaling pathway | 3.88E-07 | CXCL10; DDX58; IRF7; ISG15 |
| BioCarta | Pertussis toxin-insensitive CCR5 Signaling in Macrophage | 4.05E-04 | JUN; CCL2 |
|  | Inhibition of Cellular Proliferation by Gleevec | 9.08E-04 | JUN; STAT1 |
|  | TPO Signaling Pathway | 9.08E-04 | JUN; STAT1 |
|  | The information-processing pathway at the IFN-beta enhancer | 9.08E-04 | JUN; IRF7 |
|  | IFN gamma signaling pathway | 0.016840 | STAT1 |
|  | EGF Signaling Pathway | 0.017263 | STAT1 |
|  | IGF-1 Signaling Pathway | 0.017263 | JUN |
| Reactome | Cytokine Signaling in Immune System R-HSA-1280215 | 5.64E-10 | CXCL10; JUN; STAT1; MX2; IRF7; CCL2; ISG15; IRF9; ICAM1 |
|  | Interferon Alpha/Beta Signaling R-HSA-909733 | 1.69E-08 | STAT1; MX2; IRF7; ISG15; IRF9 |
|  | Interferon Signaling R-HSA-913531 | 1.95E-08 | STAT1; MX2; IRF7; ISG15; IRF9; ICAM1 |
|  | Immune System R-HSA-168256 | 2.61E-08 | CXCL10; JUN; STAT1; MX2; IRF7; CCL2; ISG15; TLR4; IRF9; ICAM1 |
|  | Interferon Gamma Signaling R-HSA-877300 | 3.35E-06 | STAT1; IRF7; IRF9; ICAM1 |
|  | Interleukin-10 Signaling R-HSA-6783783 | 4.10E-05 | CXCL10; CCL2; ICAM1 |
|  | Signaling By Interleukins R-HSA-449147 | 4.88E-05 | CXCL10; JUN; STAT1; CCL2; ICAM1 |
|  | ISG15 Antiviral Mechanism R-HSA-1169408 | 1.45E-04 | STAT1; MX2; ISG15 |
|  | Antiviral Mechanism By IFN-stimulated Genes R-HSA-1169410 | 1.75E-04 | STAT1; MX2; ISG15 |
|  | MyD88 Dependent Cascade Initiated on Endosome R-HSA-975155 | 2.41E-04 | JUN; IRF7; TLR4 |
| WikiPathways | Non-Genomic Actions of 1 25 Dihydroxy vitamin D3 WP4341 | 2.66E-08 | JUN; STAT1; CCL2; ISG15; TLR4 |
|  | Type I Interferon Induction and Signaling During SARS-CoV-2 Infection WP4868 | 7.00E-08 | STAT1; IRF7; TLR4; IRF9 |
|  | Measles Virus Infection WP4630 | 2.19E-07 | JUN; STAT1; IRF7; TLR4; IRF9 |
|  | SARS-CoV-2 Innate Immunity Evasion and Cell Specific Immune Response WP5039 | 1.06E-06 | CXCL10; STAT1; IRF7; CCL2 |
|  | Network Map of SARS-CoV-2 Signaling Pathway WP5115 | 1.66E-06 | CXCL10; JUN; STAT1; CCL2; IRF9 |
|  | Platelet Mediated Interactions with Vascular and Circulating Cells WP4462 | 1.90E-06 | CCL2; TLR4; ICAM1 |
|  | P53 Transcriptional Gene Network WP4963 | 2.60E-06 | CCL2; ISG15; IRF9; ICAM1 |
|  | Spinal Cord Injury WP2431 | 6.62E-06 | CXCL10; CCL2; TLR4; ICAM1 |
|  | Interactions Between Immune Cells and microRNAs In Tumor Microenvironment WP4559 | 6.62E-06 | CXCL10; CCL2; TLR4 |
|  | Host Pathogen Interaction of Human Coronaviruses Interferon Induction WP4880 | 9.18E-06 | JUN; STAT1; IRF9 |
