# Supplementary material for: In-silico discovery of common molecular signatures for which SARS-CoV-2 infections and lung diseases stimulate each other, and drug repurposing
Source: PLoS One. 2024 Jul 18;19(7):e0304425. doi: 10.1371/journal.pone.0304425 (PMC11257407; doi:10.1371/journal.pone.0304425)
Supplement: S6 Table — (DOCX) [file pone.0304425.s006.docx]

**S6 Table.** List of 27 published articles associated with SARS-CoV-2 infections with their identified hub-genes.

| **Literature** | **hub-genes** | **Common hub- genes with at least 4 literatures** | **Common hub- genes with at least 3 literatures** | **Common hub- genes with at least 2 literatures** |
| --- | --- | --- | --- | --- |
| [1] | TP53, HRAS, MAPK11, RELA, IKZF3, SKI, TNFRSF13C, JAK1, TRAF6, KLRF2, CD1A, CCL5, IFNAR2, JAK2, MX1, STAT1, BID, CD55, CD80, HAL-B, and HLA-DMA | NFKB1, CCL2, MMP9, IL1B, CXCL1, IL6, ICAM1, JUN. | RELA, NFKB1, CCL2, MMP9, STAT1, CCL20, S100A12, IL1B, TLR2, IRF7, EGF, CXCL2, IL6, CXCL1, ICAM1, JUN. | TP53, RELA, CCL5, MX1, STAT1, CXCL2, TNF, NFKBIA, CSF2, TNFAIP3, CXCL1, CXCL3, CCL20, ICAM1, ISG15, JUN, AURKB, KIF11, CCNB1, CDC6, SAA2, S100A9, S100A8, SAA1, S100A12, HMOX1, FGF2, VEGFA, FOS, CCL2, NFKB1*,*IL1B, MMP9, TLR2, CXCL8, EGF, CXCL10, MMP1, SLC6A20, IFITM1, SOCS3, BIRC3, IRF7, IFITM3, ATM, IL6 |
| [2] | CXCL1, CXCL2, TNF, NFKBIA, CSF2, TNFAIP3, IL6, CXCL3, CCL20 and ICAM1 |  |  |  |
| [3] | CBL, ISG15, NEDD4, PML, REL, CTNNB1, ERBB2, JUN, RPS8 and STUB1 |  |  |  |
| [4] | PLK1, AURKB, AURKA, CDK1, CDC20, KIF11, CCNB1, KIF2C, DTL and CDC6 |  |  |  |
| [5] | FLOC, DYNLL1, FBXL3, FBXW11 |  |  |  |
| [6] | SAA2, S100A9, S100A8, SAA1 and S100A12 |  |  |  |
| [7] | HMOX1, DNMT1, PLAT, GDF1, ITGB1, ORF3a, ORF6, ORF7a, ORF7b, and ORF8 |  |  |  |
| [8] | NOTCH4, FLNC, IHH, FOSL1, CXCR4, PSMB8, DAXX, RASD2, EPN3, DIRAS1, BATF, GDF5, RGS4, CD28 |  |  |  |
| [9] | FGF2, JUN, TLR4, and VEGFA |  |  |  |
| [10] | IL6, JUN, FOS, CCL2, EGR1, NFKB1, ICAM1, NFKBIA, ATF3 and CXCL1 |  |  |  |
| [11] | IL6*,*TNF*,*IL8*,*VEGFA*,*IL1B*,*MMP9*,*STAT1*,*TLR1*,*CXCL1*,*ICAM1*,*TLR2, and IRF7 |  |  |  |
| [12] | IL6, MMP9, IL1B, CXCL8, ICAM1, FGF2, EGF, CXCL10, CCL2, CCL5, CXCL1, and FN1 |  |  |  |
| [13] | ACE2, PIR, ADH7, AKR1C2, AKR1C3 |  |  |  |
| [14] | AGTR1, CCL2, CFH, CYP2D6, CYP3A4, GSTM1, GSTP1, HAMP, HLA-B, HLA-DRB1, IFNA1, MMP1, SERPINA1, and TIMP2 |  |  |  |
| [15] | SLC6A20, LZTFL1, CCR9, FYCO1, CXCR6 and XCR1 |  |  |  |
| [16] | SLC6A20, ERMP1, FCER1G and CA11 |  |  |  |
| [17] | FGA, FGG, F5, MTHFR, KLKB1, KNG1, F2, F8 and F11 |  |  |  |
| [18] | MYC, Met, JUN, Fos, B-Raf, FGF, EGF and VEGF |  |  |  |
| [19] | ADAM8*,*C3*,*CCL20*,*CXCL14*,*FAM167A*,*METTL7A*,*SAA2*,*TYMP, CYP4F3*,*MAP7D2*,* NANOS1*,*PPARGC1A*,* VTCN1, IFITM1*,* MX2*,* SOCS3, TLR2, BIRC3, HELZ2, IRF7, MMP13, NFKB1, LIF, FOXC1, GATA2, YY1, PPARG, RELA, USF2, JUN, CREB1 and E2F1 |  |  |  |
| [20] | NELFE, RELA, KAT2A and NFKB1. |  |  |  |
| [21] | RNASE2, HP, ELANE, OLFM4, MPO, RETN, MMP8, MMP9, S100A8, S100A9, S100A12, G0S2, CDC6, CDC25A, IFN, IFI27, IFITM3, SIGLEC1 (CD169), SOCS3, ARG1 (Arginase), NELL2, RORC, KLRB1, TCF7 (TCF1), RCAN3 (Calcipressin-3), BACH2, or LEF1, IL10, CD274 |  |  |  |
| [22] | SMAD4, GSK3B, SIRT1, ATM, RIPK1, PRKACB, MED17, CCT2, BIRC3, ETS1 and TXN |  |  |  |
| [23] | ACTB, AKT1, ATM, ATP6AP1, CSNK2A1, CDK2, EGF, MAPK14, MTOR and TP53. |  |  |  |
| [24] | TPX2, DLGAP5, NCAPG, CCNB1, KIF11, HJURP, AURKB, BUB1B, TTK, and TOP2A |  |  |  |
| [25] | MX1, CCL2, CXCL10, TYROBP, STAT1, S100A12, IRF7, IL1B, TREM1, SPI1, UBE2L6, IFI44L, XAF1, IRF9, EPSTI1, ISG15, OASL, IFITM1, CMPK2, IFI6, OAS2, IFITM3. HMOX1, PPIG, MPHOSPH10, GNL2, MMP1, GADD45A, UTP6, TSR1, CCND1, PRMT1, URB1 |  |  |  |
| [26] | TLR2, USP53, GUCY1A2, SNRPD2, NEDD9, IGF2, CXCL2, KLF6, PAG1 and ZFP36 |  |  |  |
| [27] | IL6, [CXCL8](https://www.sciencedirect.com/topics/biochemistry-genetics-and-molecular-biology/chemokine), [CXCL1](https://www.sciencedirect.com/topics/biochemistry-genetics-and-molecular-biology/lymphotactin), CCL20, CXCL2, CXCL3, [IL1B](https://www.sciencedirect.com/topics/biochemistry-genetics-and-molecular-biology/interleukin-1), IL1A, MMP9, CXCL5, [CSF3](https://www.sciencedirect.com/topics/biochemistry-genetics-and-molecular-biology/colony-stimulating-factor), [ICAM1](https://www.sciencedirect.com/topics/biochemistry-genetics-and-molecular-biology/intercellular-adhesion-molecule), IL1RN, CSF2, [PTGS2](https://www.sciencedirect.com/topics/biochemistry-genetics-and-molecular-biology/prostaglandin-endoperoxide-synthase-2), [SAA1](https://www.sciencedirect.com/topics/biochemistry-genetics-and-molecular-biology/serum-amyloid-a1), NFKB1, and TNFAIP3 |  |  |  |

**References**

1. Vastrad B, Vastrad C, Tengli A. Identification of potential mRNA panels for severe acute respiratory syndrome coronavirus 2 (COVID-19) diagnosis and treatment using microarray dataset and bioinformatics methods. 3 Biotech. 2020;10(10):1-65.

2. Xie TA, Han MY, Su XR, Li HH, Chen JC, Guo XG. Identification of Hub genes associated with infection of three lung cell lines by SARS‐CoV‐2 with integrated bioinformatics analysis. Journal of cellular and molecular medicine. 2020;24(20):12225.

3. Vastrad B, Vastrad C, Tengli A. Bioinformatics analyses of significant genes, related pathways, and candidate diagnostic biomarkers and molecular targets in SARS-CoV-2/COVID-19. Gene Reports. 2020;21:100956.

4. Auwul MR, Rahman MR, Gov E, Shahjaman M, Moni MA. Bioinformatics and machine learning approach identifies potential drug targets and pathways in COVID-19. Briefings in bioinformatics. 2021;22(5):bbab120.

5. Gu H, Yuan G. Identification of key genes in SARS-CoV-2 patients on bioinformatics analysis. bioRxiv. 2020.

6. Taz TA, Ahmed K, Paul BK, Al-Zahrani FA, Mahmud SH, Moni MA. Identification of biomarkers and pathways for the SARS-CoV-2 infections that make complexities in pulmonary arterial hypertension patients. Briefings in Bioinformatics. 2021;22(2):1451-65.

7. Sardar R, Satish D, Gupta D. Identification of novel SARS-CoV-2 drug targets by host microRNAs and transcription factors co-regulatory interaction network analysis. Frontiers in genetics. 2020;11:571274.

8. Mahmud SH, Al-Mustanjid M, Akter F, Rahman MS, Ahmed K, Rahman MH, et al. Bioinformatics and system biology approach to identify the influences of SARS-CoV-2 infections to idiopathic pulmonary fibrosis and chronic obstructive pulmonary disease patients. Briefings in Bioinformatics. 2021;22(5):bbab115.

9. Ceylan H. A bioinformatics approach for identifying potential molecular mechanisms and key genes involved in COVID-19 associated cardiac remodeling. Gene Reports. 2021;24:101246.

10. Chen W, Fang Z, Lv X, Zhou Q, Yao M, Deng M. Prediction of potential therapeutic drugs against SARS-CoV-2 by using Connectivity Map based on transcriptome data. Eur Rev Med Pharmacol Sci. 2021;25:3122-31.

11. Alsamman AM, Zayed H. The transcriptomic profiling of SARS-CoV-2 compared to SARS, MERS, EBOV, and H1N1. PLoS One. 2020;15(12):e0243270.

12. Fang K-Y, Cao W-C, Xie T-A, Lv J, Chen J-X, Cao X-J, et al. Exploration and validation of related hub gene expression during SARS-CoV-2 infection of human bronchial organoids. Human Genomics. 2021;15(1):1-13.

13. Heng-Yi Y, Mao T-N. Starting from the Influence of Tobacco: AKR1C3 and COVID-19 Receptor ACE2 Are Potential Pprognostic Biomarkers for Brain Lower Grade Glioma, Evidence from Bioinformatics Analyses. Preprints. 2020.

14. Dolan ME, Hill DP, Mukherjee G, McAndrews MS, Chesler EJ, Blake JA. Investigation of COVID-19 comorbidities reveals genes and pathways coincident with the SARS-CoV-2 viral disease. Scientific reports. 2020;10(1):1-11.

15. Group SC-G. Genomewide association study of severe Covid-19 with respiratory failure. New England Journal of Medicine. 2020;383(16):1522-34.

16. Hernandez Cordero A, Li X, Milne S, Yang C, Bossé Y, Joubert P, et al. Integrative genomic analysis highlights potential genetic risk factors for Covid-19. TP91 TP091 EPIDEMIOLOGY AND TRANSLATIONAL ADVANCES IN SARS-COV-2: American Thoracic Society; 2021. p. A3765-A.

17. Abu-Farha M, Al-Sabah S, Hammad MM, Hebbar P, Channanath AM, John SE, et al. Prognostic genetic markers for thrombosis in COVID-19 patients: a focused analysis on D-dimer, homocysteine and thromboembolism. Frontiers in pharmacology. 2020;11:587451.

18. Souchelnytskyi S, Nera A, Souchelnytskyi N. COVID-19 engages clinical markers for the management of cancer and cancer-relevant regulators of cell proliferation, death, migration, and immune response. Scientific reports. 2021;11(1):1-11.

19. Nain Z, Barman SK, Sheam MM, Syed SB, Samad A, Quinn JM, et al. Transcriptomic studies revealed pathophysiological impact of COVID-19 to predominant health conditions. Briefings in Bioinformatics. 2021;22(6):bbab197.

20. Fujisawa K, Shimo M, Taguchi Y-H, Ikematsu S, Miyata R. PCA-based unsupervised feature extraction for gene expression analysis of COVID-19 patients. Scientific reports. 2021;11(1):1-11.

21. Aschenbrenner AC, Mouktaroudi M, Krämer B, Oestreich M, Antonakos N, Nuesch-Germano M, et al. Disease severity-specific neutrophil signatures in blood transcriptomes stratify COVID-19 patients. Genome medicine. 2021;13(1):1-25.

22. Ahmed FF, Reza MS, Sarker MS, Islam MS, Mosharaf MP, Hasan S, et al. Identification of host transcriptome-guided repurposable drugs for SARS-CoV-1 infections and their validation with SARS-CoV-2 infections by using the integrated bioinformatics approaches. PloS one. 2022;17(4):e0266124.

23. Siminea N, Popescu V, Sanchez Martin JA, Florea D, Gavril G, Gheorghe A-M, et al. Network analytics for drug repurposing in COVID-19. Briefings in bioinformatics. 2022;23(1):bbab490.

24. Hasan MI, Rahman MH, Islam MB, Islam MZ, Hossain MA, Moni MA. Systems Biology and Bioinformatics approach to Identify blood based signatures molecules and drug targets of patient with COVID-19. Informatics in Medicine Unlocked. 2022;28:100840.

25. Chen Q, Xia S, Sui H, Shi X, Huang B, Wang T. Identification of hub genes associated with COVID-19 and idiopathic pulmonary fibrosis by integrated bioinformatics analysis. PloS one. 2022;17(1):e0262737.

26. Mosharaf M, Reza M, Kibria M, Ahmed FF, Kabir M, Hasan S, et al. Computational identification of host genomic biomarkers highlighting their functions, pathways and regulators that influence SARS-CoV-2 infections and drug repurposing. Scientific reports. 2022;12(1):1-22.

27. El-Aarag SA, Mahmoud A, ElHefnawi M. Identifying potential novel insights for COVID-19 pathogenesis and therapeutics using an integrated bioinformatics analysis of host transcriptome. International journal of biological macromolecules. 2022;194:770-80.
