# Supplementary material for: In-silico discovery of common molecular signatures for which SARS-CoV-2 infections and lung diseases stimulate each other, and drug repurposing
Source: PLoS One. 2024 Jul 18;19(7):e0304425. doi: 10.1371/journal.pone.0304425 (PMC11257407; doi:10.1371/journal.pone.0304425)
Supplement: S7 Table — (DOCX) [file pone.0304425.s007.docx]

**S7 Table.** List of 27 published articles associated with lung disease with their identified hub-genes.

| **Literature** | **hub-genes** | **Common hub-genes with at least 4 literatures** | **Common hub-genes with at least 3 literatures** | **Common hub-genes with at least 2 literatures** |
| --- | --- | --- | --- | --- |
| [1] | NOTCH4, FLNC, Indian hedgehog (IHH), FOSL1, CXCR4, PSMB8, DAXX, RASD2, EPN3, DIRAS1, BATF, GDF5, RGS4, and CD28 | IL6, SPP1, VCAM1, MMP7, CXCL14, MMP1 | FGG, SFTPD, IL6, COL1A2, SPP1, VCAM1, CXCL14, MMP7, SOCS3, MMP1. | CD28, GPR15, SFXN3, CYP1B1, VEGFA, CX3CR1, PPBP, CXCL8, IL1R1, SFTPC, GREM1,  CXCL1, CXCL13, CXCL14, SPP1, SPARC, ARRB1, EP300, CUL3, COL14A1, COL15A1, TNC, JUN, COL17A1, SULF1, COL1A1, POSTN, COL10A1, ACADL, EDNRB, HHIP, CXCR2, CXCL12, SOCS3, SERPINE1, CCND1, MYC, IGF1, IL4, FGG, SFTPD, IL6, COL1A2, VCAM1, MMP7, MMP1, CDH1, ICAM1, CTGF. |
| [2] | BCORP1, EIF1AY, KDM5D, MAP7D2, PRKY, RPS4Y1, TTTY10, TTTY14, USP9Y, CCR8, FAS and CASP9, MDM2, CyclinD1, ECM, DUSP7, GPR15, PLD1, FICD, CBLL1, FCGR1B, TCF7, LPAL2, SFXN3, RPL10L, DDX3Y, and DLEU7 |  |  |  |
| [3] | CYP1B1, VEGFA, BCL2, and CDKN1A |  |  |  |
| [4] | CX3CR1, PPBP, PTGS2, FPR1, FPR2, S100A12, ARG1, EGR1, CD163,  VCAM1, FGG, ORM1, S100A8 and S100A9 |  |  |  |
| [5] | IL6, CXCL8 (IL8), CYC1, ATP5C1, NDUFA12, C1QBP, ATP5A1, SDHB, ATP5O, ECH1, ACADVL, and SFXN3, TNF (TNF α), and CSF2 (GM-CSF), TLR2, 14 TLR4, and IL1R1, FGA, FGG, SFTPD |  |  |  |
| [6] | FGG, MCL1, PDE4A, S100A6, IL6, SFTPC, TMSB4X, PSMA2, PSMB1, PSMC5, PSMD4, HNRNPH1, VIM, PSMD13, STUB1, SELS, and DERL2 |  |  |  |
| [7] | ACER2, LMAN2L, CHRM3, SLIT2, HDAC10 and STOML2, HDAC2, BAX, ROS, CELSR1 and IFI27 |  |  |  |
| [8] | ATG3, CHN2, DPYS, GAS2, GGN, CRIP1, DPP6, FREM3, GALNT14, QRFPR, EVA1C, GAD1, MOB3C, PLCG2, RAC1, SELENOH, URB1, WDR4, C10orf53, CRISP3, ITM2A, PEX5L, RIPOR3, SCGB3A2, SFTPB |  |  |  |
| [9] | CYP1A1, CYP1B1, AHRR, CLDN10, TMEM2, ALDH1A3, APOD, CYP2A6, SCGB1A1, FCN1, CD28, GPR15 |  |  |  |
| [10] | CCL15, RPGRIP1, DNAH6, DNAH7, DNAI1, MUC5B, GREM1, MMP7, FHL2*,* CTHRC1, XCL1, CCL17, CCL5, CXCL9, CXCL10, CXCL11, CXCL1, CXCL6, CCL7, CXCL13, CCL14, CXCL14, FOXJ1, NELL2, SCGB3A1, LRRC34, MYL3, MMP1, AGER, DEFA3, COL1A2, CCNA2, CCR2, ITGAM and SNTN |  |  |  |
| [11] | RAB11FIP1, TGFBR3, SPP1, HLA-DOA, DOB, DPA1, DPB1, DQA1, DQB1, DQB2, DRA, DRB1, DRB3, SPARC, HPS3, SFTPD, SFTPC, SFTPA1, SFTPA2, MMP7, GPX8, VCAM1, ARRB1, HLA-B, LDLR, GABARAPL1, SGTA |  |  |  |
| [12] | EP300*,*MMP2*,*CDH2*,*CDK2*,*GNG10*,*ALB*,*SMC2*,*DHX15*,*CUL3*,*BTBD1*,* and LTN1 |  |  |  |
| [13] | SFRP2, COL14A1, COL15A1, MMP11, SPP1, LOXL1, MMP1, TNC, ITGA11, COL17A1, COL10A1, MFAP2, VCAM1, FBLN2, SULF1, FAP, COL1A1, MMP13, CCDC80, GREM1, TTR, POSTN, MMP7, MMP10, COMP. P2RY6, NTS, ADRB1, VIPR1, RXFP1, EDNRE, BDKRB2, CXCL14, DAPL1, DOK5, FNDC4. |  |  |  |
| [14] | MMP7, TRIM2, ASPN, SULF1, CXCL14, DCLK1, IL13RA2, TP63, CRTAC1, COL17A1, COL3A1, COL6A3, COL1A2, COL15A1, COL1A1, COL5A2, COL10A1, TNC, COL14A1, TSHZ2, IL1R2 and SLCO4A1. |  |  |  |
| [15] | ACADL, CD36, LPL, MMP1, HSD17B6, RRM2, EDNRB, HHIP, AOC3, MME, CA2, CA4, SFTPD, SCN7A, TNNC1, CXCL14, CXCR2 |  |  |  |
| [16] | CXCL12, CXCL14, LTBP1, TGFB3, ATF3, HHIP, BOC, NBL1, SOCS3, SERPINE1, CXCR2, Tenasein C, FABP4, CCND1, ACADL, HMGCR-FLT1-FZD5-ARRB1 |  |  |  |
| [17] | IL-8, TNF-a, MCP-1, BALF, SOD, CAT, mTOR, NEC-1, KNG1, EDNRB |  |  |  |
| [18] | IL6, CXCL8, MMP9, SNAI1, CYCS, HIF1A, SPP1, THBS1, MMP1, POSTN, CXCL13, CD86, CD19, SDC1, HMOX1, ATM, CX3CR1, GNG13, ANGPT1 and TNFRSF1A |  |  |  |
| [19] | MYC, VEGFA, MAPK8, STAT5A, FOXO3, FOXO1, IGF1, NR3C1, HSPA8, and EP300 FBXW7, CUL3, BTRC, ZBTB16, ANAPC13, CDC34, FBXL18, FBXL20, SMURF1, and UBE2V1 |  |  |  |
| [20] | SOCS3, IL4, IL18R1, IL1R1, and IL6 |  |  |  |
| [21] | FBN1, ADD3, AHNAK, AP1G1, BAZ2A, BTG2, TMEM9B, ELK4, ACTB, MARCKS, YWHAG, SLC4A4, ARFGEF2, BIR3BP, ZNF423, KDM5A, USP37, CCNG1, CCND1, CELF1, DDX3X, MAP1B, MYC, CTNNB1, CDH1 and NUFIP1 |  |  |  |
| [22] | JUN, FOS, STAT3, SOCS3, JUNB, DUSP1, IL4, FCER1A, MS4A2, and CPA3 |  |  |  |
| [23] | PPBP, MAPK, SPP1 |  |  |  |
| [24] | ADCY8, CRH, FGB, GPR17, MCHR1, NMUR1, and SAA1 |  |  |  |
| [25] | ICAM1, CDH1, CXCL12, JUN, CTGF, SERPINE1, CXCL1, EDN1, COL1A2, VCAM1 and SPARC |  |  |  |
| [26] | IFIT2, IFIT3, RSAD2, and PARP14 |  |  |  |
| [27] | CTGF, CCL2, IGF1, EGFR and ICAM1 |  |  |  |

**References**

1. Mahmud SH, Al-Mustanjid M, Akter F, Rahman MS, Ahmed K, Rahman MH, et al. Bioinformatics and system biology approach to identify the influences of SARS-CoV-2 infections to idiopathic pulmonary fibrosis and chronic obstructive pulmonary disease patients. Briefings in Bioinformatics. 2021;22(5):bbab115.

2. Rogers LR, Verlinde M, Mias GI. Gene expression microarray public dataset reanalysis in chronic obstructive pulmonary disease. Plos one. 2019;14(11):e0224750.

3. Yang D, Yan Y, Hu F, Wang T. CYP1B1, VEGFA, BCL2, and CDKN1A affect the development of chronic obstructive pulmonary disease. International Journal of Chronic Obstructive Pulmonary Disease. 2020;15:167.

4. Huang X, Li Y, Guo X, Zhu Z, Kong X, Yu F, et al. Identification of differentially expressed genes and signaling pathways in chronic obstructive pulmonary disease via bioinformatic analysis. FEBS Open Bio. 2019;9(11):1880-99.

5. Ham S, Oh Y-M, Roh T-Y. Evaluation and interpretation of transcriptome data underlying heterogeneous chronic obstructive pulmonary disease. Genomics & informatics. 2019;17(1).

6. Kim WJ, Lim JH, Lee JS, Lee S-D, Kim JH, Oh Y-M. Comprehensive analysis of transcriptome sequencing data in the lung tissues of COPD subjects. International journal of genomics. 2015;2015.

7. Jeong I, Lim J-H, Oh DK, Kim WJ, Oh Y-M. Gene expression profile of human lung in a relatively early stage of COPD with emphysema. International Journal of Chronic Obstructive Pulmonary Disease. 2018;13:2643.

8. Roessler FK, Benedikter BJ, Schmeck B, Bar N. Novel computational analysis of large transcriptome datasets identifies sets of genes distinguishing chronic obstructive pulmonary disease from healthy lung samples. Scientific reports. 2021;11(1):1-13.

9. Morrow JD, Chase RP, Parker MM, Glass K, Seo M, Divo M, et al. RNA-sequencing across three matched tissues reveals shared and tissue-specific gene expression and pathway signatures of COPD. Respiratory research. 2019;20(1):1-12.

10. Karman J, Wang J, Bodea C, Cao S, Levesque MC. Lung gene expression and single cell analyses reveal two subsets of idiopathic pulmonary fibrosis (IPF) patients associated with different pathogenic mechanisms. PloS one. 2021;16(3):e0248889.

11. Qian W, Cai X, Qian Q, Zhang X. Identification and Validation of Potential Biomarkers and Pathways for Idiopathic Pulmonary Fibrosis by Comprehensive Bioinformatics Analysis. BioMed Research International. 2021;2021.

12. Qiu X, Lin J, Liang B, Chen Y, Liu G, Zheng J. Identification of hub genes and microRNAs associated with idiopathic pulmonary arterial hypertension by integrated bioinformatics analyses. Frontiers in genetics. 2021:544.

13. Chen S, Zhang J, Ma W, Ye H. Identification of differentially expressed genes triggered by aberrant methylation in idiopathic pulmonary fibrosis using integrated bioinformatic analysis. 2021.

14. Li D, Liu Y, Wang B. Identification of transcriptomic markers for developing idiopathic pulmonary fibrosis: An integrative analysis of gene expression profiles. International journal of clinical and experimental pathology. 2020;13(7):1698.

15. Leng D, Yi J, Xiang M, Zhao H, Zhang Y. Identification of common signatures in idiopathic pulmonary fibrosis and lung cancer using gene expression modeling. BMC cancer. 2020;20(1):1-15.

16. Gangwar I, Kumar Sharma N, Panzade G, Awasthi S, Agrawal A, Shankar R. Detecting the molecular system signatures of idiopathic pulmonary fibrosis through integrated genomic analysis. Scientific reports. 2017;7(1):1-11.

17. Shi K, Chen X, Xie B, Yang SS, Liu D, Dai G, et al. Celastrol alleviates chronic obstructive pulmonary disease by inhibiting cellular inflammation induced by cigarette smoke via the Ednrb/Kng1 signaling pathway. Frontiers in pharmacology. 2018;9:1276.

18. Lin Y-Z, Zhong X-N, Chen X, Liang Y, Zhang H, Zhu D-L. Roundabout signaling pathway involved in the pathogenesis of COPD by integrative bioinformatics analysis. International journal of chronic obstructive pulmonary disease. 2019;14:2145.

19. Zhu M, Ye M, Wang J, Ye L, Jin M. Construction of Potential miRNA–mRNA Regulatory Network in COPD Plasma by Bioinformatics Analysis. International journal of chronic obstructive pulmonary disease. 2020;15:2135.

20. Chen W, Hong Y, Meng Z. Bioinformatics analysis of molecular mechanisms of chronic obstructive pulmonary disease. Eur Rev Med Pharmacol Sci. 2014;18(23):3557-63.

21. Liu X, Qu J, Xue W, He L, Wang J, Xi X, et al. Bioinformatics-based identification of potential microRNA biomarkers in frequent and non-frequent exacerbators of COPD. International Journal of Chronic Obstructive Pulmonary Disease. 2018;13:1217.

22. Liu L, Cai D, Wu Y. Bioinformatics Analysis Identifies Potential Key Genes of Peripheral Blood Mononuclear Cell in Idiopathic Pulmonary Fibrosis. Age (years). 2020;65(10.63):69.00-8.16.

23. Wang H, Wang M, Xiao K, Zhang X, Wang P, Xiao S, et al. Bioinformatics analysis on differentially expressed genes of alveolar macrophage in IPF. Experimental lung research. 2019;45(9-10):288-96.

24. Mishra S, Shah MI, Kumar SU, Kumar DT, Gopalakrishnan C, Al-Subaie AM, et al. Network analysis of transcriptomics data for the prediction and prioritization of membrane-associated biomarkers for idiopathic pulmonary fibrosis (IPF) by bioinformatics approach. Advances in protein chemistry and structural biology. 2021;123:241-73.

25. Xu Z, Mo L, Feng X, Huang M, Li L. Using bioinformatics approach identifies key genes and pathways in idiopathic pulmonary fibrosis. Medicine. 2020;99(36).

26. Zheng J-N, Li Y, Yan Y-M, Shi H, Zou T-T, Shao W-Q, et al. Identification and validation of key genes associated with systemic sclerosis-related pulmonary hypertension. Frontiers in Genetics. 2020;11:816.

27. Lu Y, Li A, Lai X, Jiang J, Zhang L, Zhong Z, et al. Identification of differentially expressed genes and signaling pathways using bioinformatics in interstitial lung disease due to tyrosine kinase inhibitors targeting the epidermal growth factor receptor. Investigational New Drugs. 2019;37(2):384-400.
