# Supplementary figures and images for: In-silico discovery of common molecular signatures for which SARS-CoV-2 infections and lung diseases stimulate each other, and drug repurposing
Source: PLoS One. 2024 Jul 18;19(7):e0304425. doi: 10.1371/journal.pone.0304425 (PMC11257407; doi:10.1371/journal.pone.0304425)

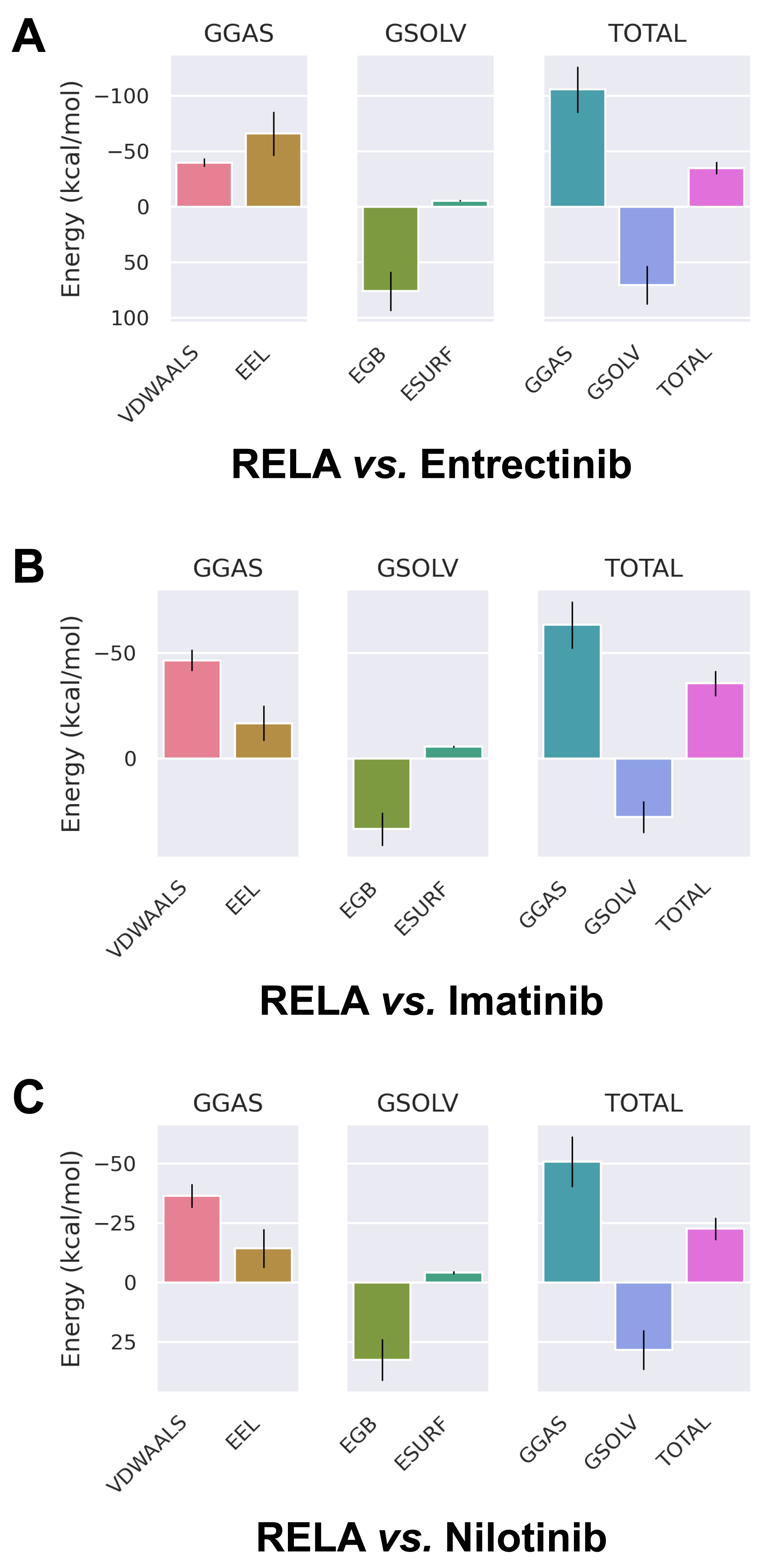

Supplement: S1 Fig — (TIF) [file pone.0304425.s012.tif]
